# Impact of Cleaning on Membrane Performance during Surface Water Treatment: A Hybrid Process with Biological Ion Exchange and Gravity-Driven Membranes

**DOI:** 10.3390/membranes14020033

**Published:** 2024-01-25

**Authors:** Yaser Rasouli, Benoit Barbeau, Raphaël Maltais-Tariant, Caroline Boudoux, Dominique Claveau-Mallet

**Affiliations:** 1 Department of Civil, Geological & Mining Engineering, Polytechnique Montréal, 2900 Boulevard Édouard-Montpetit, Montréal, QC H3T 1J4, Canada; yaser.rasouli@polymtl.ca (Y.R.); benoit.barbeau@polymtl.ca (B.B.); 2 Department of Engineering Physics, Polytechnique Montréal, 2900 Édouard-Montpetit, Montréal, QC H3T 1J4, Canada; raphael.maltais-tariant@polymtl.ca (R.M.-T.); caroline.boudoux@polymtl.ca (C.B.); 3 Castor Optics, Inc., St-Laurent, QC H3T 1J4, Canada

**Keywords:** hydraulic reversible fouling, flux recovery, permeate quality, membrane cleaning, polymeric membranes, ceramic membranes

## Abstract

In this study, the hybrid biological ion exchange (BIEX) resin and gravity-driven membrane (GDM) process was employed for the treatment of coloured and turbid river water. The primary objective was to investigate the impact of both physical and chemical cleaning methods on ceramic and polymeric membranes in terms of their stabilised flux, flux recovery after physical/chemical cleaning, and permeate quality. To address these objectives, two types of MF and UF membranes were utilised (M1 = polymeric MF, M2 = polymeric UF, M3 = ceramic UF, and M4 = lab-made ceramic MF). Throughout the extended operation, the resin functioned initially in the primary ion exchange (IEX) region (NOM displacement with pre-charged chloride) and progressed to a secondary IEX stage (NOM displacement with bicarbonate and sulphate), while membrane flux remained stable. Subsequently, physical cleaning involved air/water backwash with two different flows and pressures, and chemical cleaning utilised NaOH at concentrations of 20 and 40 mM, as well as NaOCl at concentrations of 250 and 500 mg Cl_2_/L. These processes were carried out to assess flux recovery and identify fouling reversibility. The results indicate an endpoint of 1728 bed volumes (BVs) for the primary IEX region, while the secondary IEX continued up to 6528 BV. At the end of the operation, DOC and UVA_254_ removal in the effluent of the BIEX columns were 68% and 81%, respectively, compared to influent water. This was followed by 30% and 57% DOC and UVA_254_ removal using M4 (ceramic MF). The stabilised flux remained approximately 3.8–5.2 LMH both before and after the cleaning process, suggesting that membrane materials do not play a pivotal role. The mean stabilised flux of polymeric membranes increased after cleaning, whereas that of the ceramics decreased. Enhanced air–water backwash flow and pressure resulted in an increased removal of hydraulic reversible fouling, which was identified as the dominant fouling type. Ceramic membranes exhibited a higher removal of reversible hydraulic fouling than polymeric membranes. Chemical cleaning had a low impact on flux recovery; therefore, we recommend solely employing physical cleaning.

## 1. Introduction

In various parts of the world, rural populations face challenges in accessing simple, cost-effective, and locally produced water treatment technologies. Unfortunately, research on water supply options for rural and remote regions is often overshadowed by the issues encountered in urban areas. Advanced and centralised technologies designed for large-scale operations and skilled personnel cannot be directly applied to smaller systems due to factors such as energy efficiency, robustness, resilience, and staffing requirements, all of which justify the exploration of alternative solutions [1,2,3]. In this context, passive decentralised water treatment systems emerge as an appealing option. They have been developed to reduce maintenance requirements, diminish the reliance on trained operators, simplify design, and allow for potential off-grid operations [4,5,6].

We recently proposed a passive decentralised treatment option comprising the biological ion exchange (BIEX) process combined with gravity-driven membrane filtration (GDM). This system demonstrated the ability to operate for over 60 days without backwash or regeneration, producing potable water using turbid (5–10 NTU) and coloured (TOC ≈ 7 mg C/L) river water [7]. The BIEX filtration process involves the operation of an anionic resin with sporadic regeneration to deplete pre-loaded anions (typically chloride). It operates in the secondary ion exchange (IEX) region, taking advantage of the self-regeneration induced by naturally occurring anions in the influent water, such as bicarbonate and/or sulphate anions [8,9]. These are displaced by NOM from the influent water. Delaying the regeneration stage reduces secondary pollution from spent brine, the transport of regeneration chemicals, maintenance, and costs [10,11]. However, the BIEX is not a robust treatment for particulate matter. The main challenges associated with the BIEX filtration process stem from the emergence of a Schmutzdecke phenomenon. This involves the formation of a difficult-to-break-up biological layer on the upper surface of the column, which may be effectively disrupted through the introduction of air injection. A DOC breakthrough during a transition from primary IEX to secondary IEX could also be considered as another issue in BIEX filtration.

GDM filtration, whether utilising ultrafiltration or microfiltration, is considered an efficient removal process. This method necessitates a simple and compact installation, fewer ancillary equipment, lower energy consumption, and operators with lower skill levels [12,13,14]. Nevertheless, the primary challenges associated with the GDM process, such as achieving a low stabilised flux and a reduction in NOM removal, can be mitigated by opting for BIEX resin as a pre-treatment method. Therefore, BIEX resin pretreatment followed by GDM filtration appears to be a competitive process for eliminating turbidity, colour, and NOM in decentralised surface water treatment applications. For such applications, selecting the right membrane type is essential, maximising productivity and permeate water quality. Comparing the permeability of polymeric and ceramic membranes, it was observed that, for a similar MWCO, polymeric membranes often exhibit higher clean water permeability than ceramic membranes due to the module design (hollow fibre vs. monolithic for ceramics) [15,16,17,18]. However, opposite results can be found in the literature. For example, in a study published by Kook et al. [19], 0.1-µm ceramic Al_2_O_3_ membrane (2440 LMH/bar) showed a substantially higher permeability than that of a 0.1 µm polymeric PVDF membranes (992–1621 LMH/bar).

Concerning the fouling tendency, some studies reported similar [20] or slightly lower [21] fouling for ceramic membranes. In general, the production cost of ceramic membranes (capital cost) exceeds that of polymeric membranes [21,22,23]. However, the higher cost of ceramic membranes can be offset by their longer lifespan, approximately 20 years, which is roughly twice that of polymeric membranes [24,25,26].

A crucial advantage of ceramic membranes is their ability to undergo aggressive chemical and physical cleaning without damaging the membrane materials [27]. For instance, Park et al. [28] studied the effect of physical and chemically enhanced backwash on the flux recovery of a 0.1 µm ceramic membranes using DI water injection at 500 kPa and 300 mg/L of NaOCl. The maximum flux recovery obtained was 100% in this study.

Given that GDM processes aim to minimise physical and chemical backwashes, the question arises whether the advantages of ceramic membranes are justified in such applications. Alresheedi et al. [29] demonstrated that backwashes were twice as effective for removing NOM fouling from a ceramic UF (SiC) membrane compared to a polymeric PVDF UF membrane. However, no differences in the removal of irreversible fouling using NaOH and NaOCl were observed between the two membrane types. To date, no equivalent evaluation has been performed to compare the cleaning performances of ceramic and polymeric membranes used in GDM filtration.

A current industry trend involves conducting more frequent yet less aggressive chemical washes to manage fouling [30]. Such a strategy, often referred to as chemically enhanced backwash, contrasts with GDM operation, where low maintenance is desired, and cake filtration is favoured to stabilise the flux. Consequently, the chemical washing strategy necessarily differs for GDM filtration systems installed in small decentralised systems. Research is needed to define the optimal chemical conditions and potential benefits of using ceramic membranes to enable aggressive chemical washes of membranes that have been operated for several months without cleaning.

In the present study, the performances of two ceramics (commercial UF and lab-made MF) and two polymeric membranes (commercial UF and MF) were compared during the treatment of turbid, coloured river water with a high NOM content using a hybrid BIEX resin + GDM filtration process. After approximately 76 days of operation, physical (air/water backwash with enhanced or decreased air/water flow and pressure) and various chemical cleaning conditions (250 or 500 mg Cl_2_/L of NaOCl; 20 or 40 mM of NaOH) were tested on the membranes to quantify the removal of different fouling types (reversible/irreversible, physical/chemical). Finally, the effects of the physical cleaning conditions (air/water backwash flow and pressure) and the impact of increasing the chemical agent concentration during chemical cleaning on the flux recovery and fouling removal of each membrane type are discussed.

## 2. Materials and Methods

### 2.1. Biological Ion Exchange Resin and Gravity-Driven Filtration Experiments

**Figure 1:**
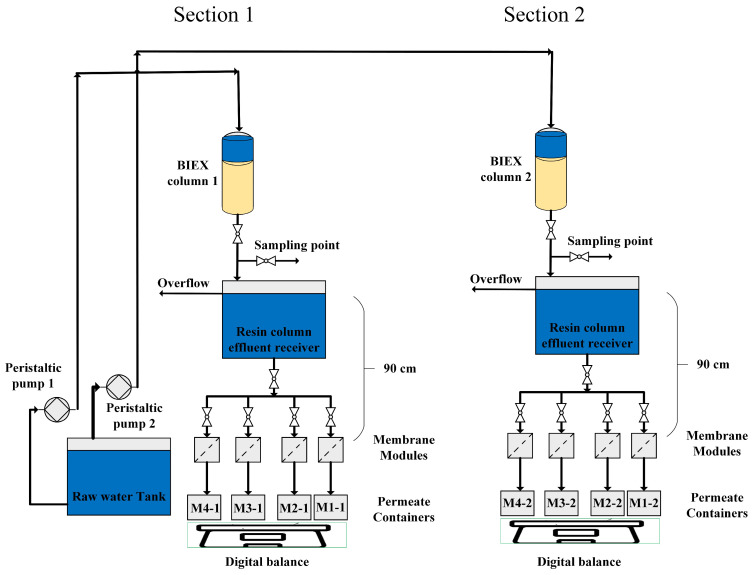
Experimental BIEX + GDM filtration pilot setup. BIEX resin operated at a 4 BV/h filtration rate and EBCT = 15 min. M1-1 and M1-2: flat-sheet disk-shaped 0.1 µm (MF) PES, M2-1, and M2-2: flat-sheet disk-shaped 0.03 µm (UF) PES, M3-1, and M3-2: flat-sheet disk-shaped 300 kDa (UF) ceramic, M4-1 and M4-2: lab-made flat-sheet disk-shaped ceramic MF membranes (Kaolin support + alumina layer).

Figure 1 illustrates the experimental setup consisting of two identical pilots (Section 1 and Section 2). Each section comprised a resin column (BIEX column 1 and BIEX column 2) and four parallel membranes. A transparent PVC column (H = 63 cm, ϕ = 1.27 cm) was filled with an approximately 42 cm bed height of resin (resin Bed Volume ≈ 52 mL). The empty bed contact time (EBCT) and filtration rate of the resin column were kept constant at 15 min and 3.47 mL/min (≈4 BV/h; linear velocity ≈ 1.64 m/h), respectively, with values chosen based on previous studies in our group. The experiment lasted approximately 76 days (≈7300 BV), which was sufficient for achieving secondary IEX (NOM exchange with bicarbonate and/or sulphate). In this study, Purolite A860^®^, a strong base anion macroporous exchange resin (pre-loaded with chloride), was used. The resin was regenerated after 6530 BV (day 68) using NaCl (100 g/L, 2 BV/h, and 1.5 h). The anions loading of the resin was calculated using Equation (1), where *q* (eq/L resin) is the cumulative anion loading on the resin, *C_in_* and *C_out_* are the concentrations of each anion in the inlet and outlet of the resin column (eq/L), *V_resin_* is the total resin volume in the column (52 mL), *Q* is the flow rate (5 L/d), $∆t=(t_{i}-t_{i-1})$ is the time between each anion and the DOC measurement (d), and *i* is the number of anion measurements during the experiment. The charge balance was calculated only for chloride, sulphate, bicarbonate, DOC, and nitrate, which were the major negatively charged compounds during the IEX.

(1) $$q=\sum_{i=1}^{28}\frac{\left({Average\;(C}_{in,\;i},{\;C}_{in,\;i-1})-{Average\;(C}_{out,\;i},{\;C}_{out,\;i-1})\right)\times Q\times \left(t_{i}-t_{i-1}\right)}{\begin{array}{c} V_{resin}\;\\ \; \end{array}}$$

Four polymeric/ceramic MF/UF membranes were used for the membrane sections. The membrane compositions are listed in Table 1. All the membranes used in this study are commercially available, except for M4 (ceramic lab-made MF), which is a flat sheet disk-shaped ceramic MF membrane made in our laboratory, as described in Section 2.1.1. The equipment in the membrane section was covered with aluminium foil to prevent algal growth. In the receiver of the resin column effluent, an overflow was placed to maintain a transmembrane pressure of 90 cm H_2_O (≈93 mbar). The temperature was maintained at 21.2 ± 0.7 °C by the laboratory air conditioning system. The flux of the membranes (in LMH) was calculated by weighing the daily permeate volume and then normalising it to 20 °C.

#### 2.1.1. M4 Membrane Synthesis

To fabricate M4 (ceramic MF), deionised (DI) water was incrementally introduced into a mixture of kaolin clay (Ward’s Science, Rochester, NY, USA; Al_2_O_3_·2SiO_2_·2H_2_O, CAS: 1332-58-7) and boric acid (10 wt%, Ward’s Science, Rochester, NY, USA; H_3_BO_3_, CAS: 10043-35-3, crystals) to make a suitable dough. Subsequently, the dough was mixed at approximately 100 rpm with a mechanical mixer for 10 min. Following this, about 25 g of the mixture was transferred to a disk-shaped mould equipped with a cap. A pressure of 7.3 MPa (Pressure Sensor Product Inc., Madison, NJ, USA) was applied to the cap to compress the mixture and form the disk-shaped membrane, using a clamp (a freezer bag was interposed between the cap and the mould to prevent adhesion during cap removal). After placing the mould on a glass plate, the cap and plastic bag were cautiously removed. Alumina powder (10 wt% of kaolin, Fisher Scientific, Switzerland; Al_2_O_3_, 40–300 μm, CAS: 1344-28-1, Mw = 101.96 g/mol) was gently poured onto one side of the formed membrane to cover the entire surface. The membrane was then extracted from the mould after drying for a minimum of 24 h at room temperature. Finally, calcination was carried out at 1100 °C for 2 h using an electric programmable furnace (Ney Vulcan, 3–550). The schematic representing the M4 membrane production steps are shown in Figure S1. Seven membranes were synthesised for each experiment. Characterisations, such as DI water flux, porosity, pore size, SEM images, and Energy Dispersive X-ray (EDX) spectra, are provided in the Supplementary Materials (Figures S2 and S3, Table S1).

### 2.2. Characteristics of Influent Water

The experiment was conducted between October and December 2022. A single raw water sample volume of 800 L was collected using 20 L buckets at the entrance of the Pont-Viau water treatment plant (Laval, QC, Canada). Raw water originates from the Des Prairies River. The water was promptly stored at 4 °C after collection. Once a week, approximately 80 L of the collected water was extracted from the refrigerator and allowed to attain room temperature (≈22 °C) for at least 5 h before adding it to the influent water tank. The physical and chemical characteristics of the influent water from the Des Prairies River are detailed in Table 2, encompassing DOC, turbidity, pH, alkalinity, UVA_254_, nitrate, sulphate, and chloride. These low uncertainties indicate relatively constant influent water characteristics during storage.

### 2.3. Physical and Chemical Cleaning of the Membranes

Physical and chemical cleaning was conducted after 30 days of the operation to investigate the dominant fouling type and assess the impact of increasing the chemical cleaning agent concentration (during chemical cleaning) and air/water backwash flow and pressure (during physical cleaning) on membrane flux recovery and the removal of different fouling types. The procedures and steps of the cleaning processes are outlined in Table 3 and Figure S4. Physical cleaning involved air and DI water backwashing. After halting the normal filtration process, the fouled membrane surfaces were turned face down (Figure S4a), followed by air backwashing in Section 1 (pilot 1) at P = 30 psi, Q = 5 L/h, and t = 2 min; in Section 2 (pilot 2) at P = 15 psi, Q = 2.5 L/h; and t = 2 min (Figure S4b,c). Subsequently, a DI water backwash was performed for 4 h under the conditions shown in Figure S4d, with back pressures of 120 and 90 cm H_2_O for Section 1 and Section 2, respectively. The membranes were then returned to their normal filtration position (Figure S4e) to measure the DI water flux (Figure S4f) and calculate the flux recovery using Equation (2).

(2) $$\%\;Flux\;recovery=\frac{J_{ac}}{J_{v}}\times 100$$

In Equation (2), *J_ac_* is the flux after cleaning (physical or chemical), and *J_v_* is the water flux of the virgin membranes, both measured at a 90 cm H_2_O pressure and T = 20 °C.

The chemical cleaning procedure commenced by washing membranes with 40 mM of NaOH (pH = 12.40) for t = 6 h, T = 21 °C, and 90 cm H_2_O pressure in Section 1 (pilot 1). For Section 2 (pilot 2), 20 mM of NaOH (pH = 12.16) was used for t = 6 h, T = 21 °C, and 90 cm H_2_O pressure (Figure S4g). After washing with NaOH, the DI water permeability was measured to calculate flux recovery (Figure S4h). Chemical cleaning was then performed by washing the membranes with NaOCl at concentrations of 500 and 250 mg Cl_2_/L in Section 1 and Section 2, respectively, both at t = 6 h and 90 cm H_2_O pressure (Figure S4i). Finally, the DI water permeability was measured to obtain flux recovery using NaOCl (Figure S4j).

The total fouling resistance (m^−1^, R) during filtration was obtained using Darcy’s law as follows:

(3) $$R_{total}=\left(\frac{∆P}{\mu_{T}\;J_{T}}\right)-R_{clean}$$

where *μ_T_* is the permeate viscosity at temperature T, *J_T_* is the permeate flux measured at temperature T, and Δ*P* is the hydrostatic pressure. $R_{clean}$ is the clean membrane resistance calculated using the clean water permeability of the virgin membranes.

Total fouling resistance consists of (1) hydraulically reversible fouling, fouling caused by cake layer formation on the membrane surface (biofilm) which is removable via backwash (air + water), (2) hydraulically irreversible fouling, fouling caused by pore blocking which is not removable via backwash (air + water), (3) Chemically reversible fouling, fouling caused by pore blocking which is removable via chemical cleaning (NaOCl and NaOH), and (4) chemically irreversible fouling, fouling which persists after chemical cleaning.

### 2.4. Analytical Methods for Water Samples

DOC in the water samples was measured after filtration through a prewashed 0.45 µm filter and analysed with a TOC analyser (Sievers M5310C, Trevose, PA, USA). To convert the concentration from mg C/L to eq/L, the charge density of DOC (NOM) was considered to be 10 meq/g C at pH 6.5 [31] for the anion charge balance. Turbidity was measured using a turbidity meter (TL2300, Hach, Loveland, CO, USA) after calibration with standard samples. The UVA_254_ was measured using a UV-visible spectrophotometer (Cary 100 Scan, Varian Inc., Palo Alto, CA, USA) on 0.45 µm filtered samples. pH was measured using a pH meter (Accumet AB 15 basic; Fisher Scientific, Waltham, MA, USA) calibrated with standard solutions. Bicarbonate concentration (alkalinity) was determined using the standard titration method with 0.01 M H_2_SO_4_ as the titrant. To measure the anions in the BIEX resin column effluent and influent water, samples were filtered through 0.45 µm syringe filters and analysed using an ion chromatography device (ICS 5000 AS-DP DIONEX, Thermo Scientific, Waltham, MA, USA).

### 2.5. Measurement of Biofilm Thickness

To study the mean biofilm/cake thickness during filtration (especially before and after membrane cleaning), optical coherence tomography (OCT) imaging was routinely performed on the membranes that were removed from the system for approximately 30 min. For each OCT image, three random locations were selected from the membranes. Raw OCT images were obtained using a Thorlabs SL1310V1 laser (Newton, NJ, USA). The mean biofilm thickness was determined using MATLAB (R2023b) and ImageJ 1.54g software (NIH, Bethesda, MD, USA). The details and conditions of the OCT imaging were previously reported [7].

### 2.6. Statistical Analysis of Data

Analysis of variance (ANOVA) was used in Microsoft Excel^®^ (Microsoft 365) and GraphPad Prism^®^ (10.0.02) software before the membrane cleaning day to investigate the effect of the membrane types on the stabilised flux. Moreover, ANOVA was performed after membrane cleaning to determine the effect of the membrane type, chemical agent concentration (in chemical cleaning), and air/water backwash flow (in physical cleaning) on the stabilised flux (as the dependent variable) achieved after the washes. The stabilised flux is defined as a flux data group (from the flux stabilisation day to the end of the operation) in which the slope of the flux–time diagram is no more statistically significant than zero.

## 3. Results and Discussions

### 3.1. Operation of the BIEX Process

**Figure 2:**
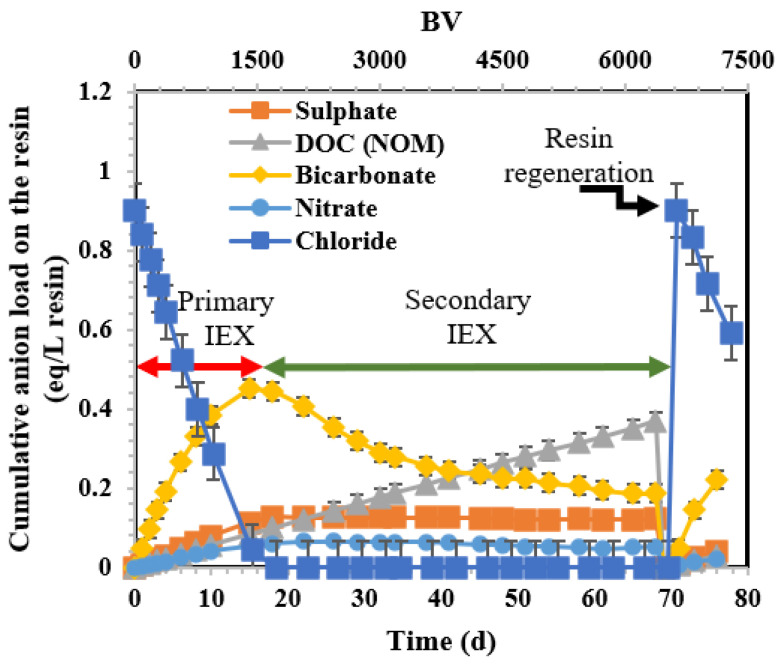
Variation in chloride, sulphate, DOC, and bicarbonate loads (as m eq/L of resin) on the BIEX 1 column during the operation. Regeneration was performed on day 68 (6528 BV). The error bars show 95% confidence intervals.

Figure 2 illustrates the calculated anion loading on the resin during the 68-day operation (6528 BV) in Column 1. The results for Column 2, depicted in Figure S5, are not discussed as they exhibit similar behaviour to those in Column 1. The primary IEX capacity of the fresh resin, approximately 0.9 eq/L of the resin, was depleted after 18 days of operation (1728 BV). At this juncture, the estimated loadings for DOC, bicarbonate, sulphate, and nitrate were 0.10, 0.45, 0.13, and 0.07 eq/L, respectively. From day 18 to day 68 (1728 to 6528 BV), the secondary IEX (BIEX mode) enabled the displacement of bicarbonate, sulphate, and nitrate anions using DOC. Consequently, the loadings for bicarbonate, sulphate, and nitrate decreased to 0.20, 0.10, and 0.04 eq/L, respectively, while NOM increased from 0.10 to 0.37 eq/L at 6528 BV. After performing regeneration on day 68 (6528 BV), the resin capacity (0.9 eq/L) was fully recovered using chloride (returning to the primary IEX region).

**Figure 3:**
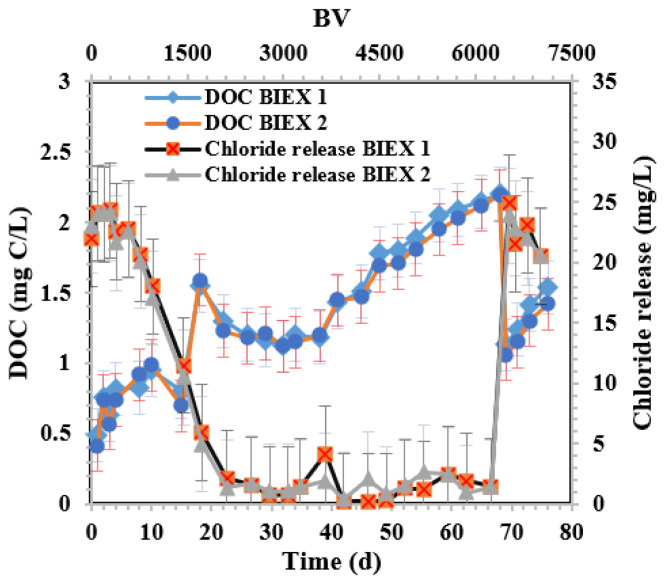
DOC concentration and chloride release in the effluent of the BIEX columns during the operation period. Day 68 (6528 BV) is the resin regeneration day. The error bars show 95% confidence intervals.

Figure 3 displays variations in effluent DOC and chloride release for BIEX-1 (BIEX column in Section 1) and BIEX-2 (BIEX column in Section 2). Both columns exhibited equivalent performance. Initially, the effluent DOC of the resin columns was 0.4 mg C/L when the chloride release from the columns was 23 mg/L. The chloride release decreased to 1.3 mg/L at approximately 1728 BV (day 18), while the DOC of the column effluent peaked at 1.55 mg C/L. Considering a constant DOC concentration in the influent water (7.04 mg C/L ± 0.18), DOC removal at the end of the primary IEX was 78%. From 1728 BV (day 18) to 6528 BV (day 68), in the secondary IEX region, DOC initially decreased due to the exchange of NOM with bicarbonate and sulphate anions. DOC remained constant from 3000 to 4000 BV and then progressively rose to 2.2 mg C/L after 6528 BV (day 68). DOC removal remained high (68%) after 68 days of the operation. At this point, the resin was regenerated, decreasing the DOC of the BIEX column effluent to 1.05 mg C/L and increasing chloride release back to 23 mg/L. Similar behaviour was observed for UVA_254_ (Figure S6a), except for the higher removal rates compared to DOC, given that humic substances have a higher affinity for the resin. The variation in the turbidity of the BIEX column effluent is shown in Figure S6b.

### 3.2. Effect of Membrane Type and Cleaning Process on the Stabilised Flux and Flux Recovery

**Figure 4:**
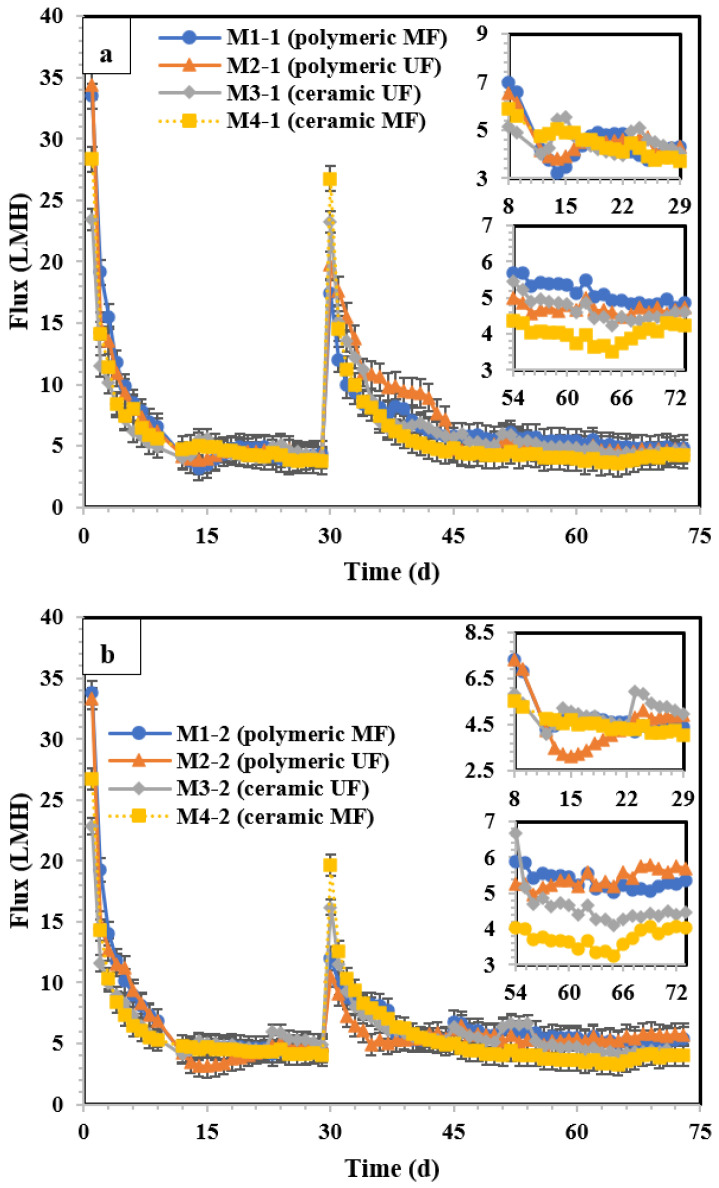
(**a**) Flux–time diagram of the membranes in (**a**) Section 1 and (**b**) Section 2. M1 (polymeric 0.1 µm MF), M2 (polymeric 0.03 µm UF), M3 (ceramic 300 kDa UF), and M4 (Lab-made ceramic MF). Day 30 is the physical and chemical cleaning day. The error bars show 95% confidence intervals.

**Figure 5:**
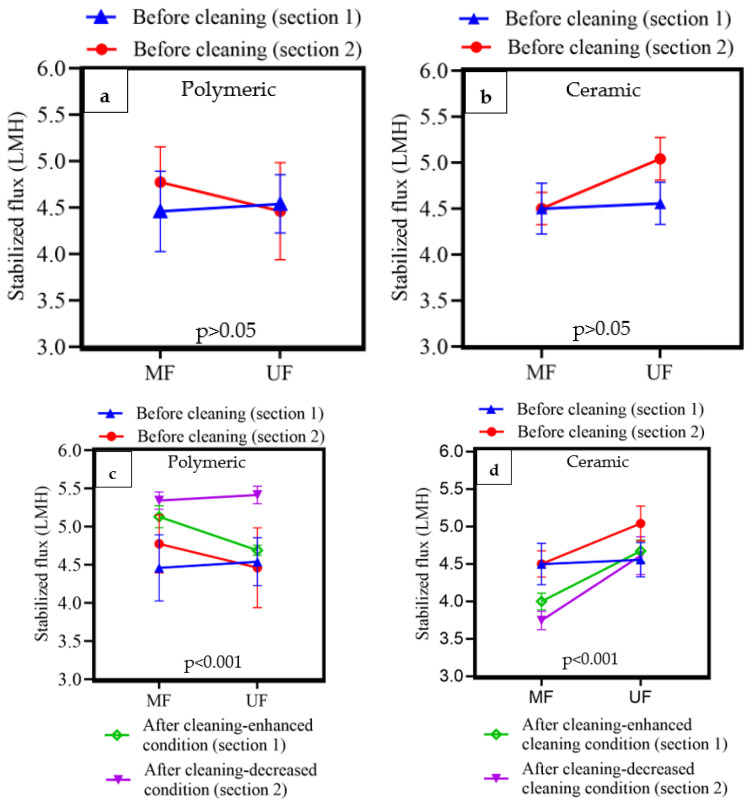
(**a**) Comparing the stabilised flux of the polymeric MF/UF membranes in Section 1 with Section 2 before the cleaning process (days 8–29). (**b**) Comparing the stabilised flux of the ceramic MF/UF membranes in Section 1 with Section 2 before the cleaning process (days 8–29). (**c**,**d**) The effect of membrane type (polymeric/ceramic, MF/UF) and the enhanced (in Section 1) or decreased cleaning condition (in Section 2) on the mean stabilised flux of the membranes after the cleaning process (day 54–73). The error bars represent 95% confidence intervals.

The variation in flux over time is shown in Figure 4. In both pilot sections before the membrane cleaning process (days 1–29), the flux stabilised at approximately day 8, after an initial steep flux decline period. To validate the stabilisation in flux, the observed flux between days 8 and 29 for each membrane type in both sections was fitted to linear regression, and the calculated slopes were not statistically higher than zero (the *p*-values of slopes for both sections were >0.05), indicating a stable operating condition. The mean stabilised fluxes of the membranes from days 8 to 29 in Section 1 and Section 2 are shown in Table 4. Prior to membrane cleaning (days 1–29), the stabilised flux was affected by the membrane type (polymeric/ceramic, MF/UF), although this difference was fairly modest. For example, the average stabilised flux from day 8 to 29 for the membranes in Section 1 and Section 2 were measured as 4.46–4.56 LMH and 4.46–5.04 LMH, respectively (Table 4). The differences in the stabilised flux from the membranes in Section 1 and Section 2 were not statistically significant (Figure 5a,b; *p* > 0.05). This implies that the cleaning conditions described in Section 1 can be compared to those described in Section 2.

After membrane cleaning (days 30–73), the flux stabilisation period was reached on approximately day 54 in both sections. Similarly, to confirm flux stabilisation, the flux from days 54 to 73 was fitted to linear regression, and the obtained slopes were not statistically different from zero (*p* > 0.05). The average stabilised flux of the membranes for days 54–73 varied between 4.00 and 5.15 LMH (in Section 1, Table 4) and 3.75–5.34 LMH (in Section 2).

After cleaning the polymeric membranes (Figure 5c), stabilised fluxes increased significantly (*p* < 0.001) from 4.46 (MF)–4.54 (UF) LMH to 5.13 (MF)–4.69 (UF) LMH (after the enhanced cleaning condition in Section 1) and from 4.77 (MF)–4.46 (UF) LMH to 5.34 (MF)–5.41 (UF) LMH (using the less stringent cleaning condition in Section 2). Although statistically significant (*p* < 0.001), increasing the cleaning intensity did not substantially improve stable fluxes from a practical perspective. The membrane type (UF vs. MF) was equally impacted by the cleaning conditions for the ceramic membranes. After cleaning the ceramic membranes (Figure 5d), the stable MF flux declined for both cleaning conditions (Section 1 and Section 2), while the UF was not impacted by cleaning in Section 1, which was subjected to enhanced cleaning conditions. In contrast, the ceramic UF in Section 2, which was subjected to a lower cleaning condition, exhibited a stabilised flux decline of 8.5%. During the clean-in-place process, some foulants were solubilised and could penetrate the membrane pores, causing internal clogging when the membranes returned to normal operation. In summary, cleaning the membranes generally led to a fairly modest increase in the stable flux, except for ceramic MF membranes.

The flux recoveries and foulant reversibility after physical and chemical washes are shown in Table 5 and Table 6, respectively. According to Table 5, most membrane fouling was hydraulically reversible (57–80%). The additional recoveries provided by cleaning with NaOH or NaOCl were modest, typically less than 10%.

The membranes in Section 1 with enhanced air/water backwash flow and pressure showed higher flux recoveries (i.e., hydraulic reversible fouling removal, 67–79%) than those in Section 2 (57–75%), which were subjected to a less intense air/water backwash flow and pressure (Table 6). The foulants were more hydraulically reversible on the ceramic membranes (70–79%) than on the polymeric membranes (57–69%). These findings suggest that it is simpler to remove foulants from ceramic membranes than from polymeric membranes using a basic backwash method involving air and water. The foulants left on the polymeric membranes after physical cleaning were difficult to remove via chemical cleaning. On average, 26% of the foulants were chemically irreversible. In contrast, the ceramic membranes had the lowest fraction of irreversible fouling (13%), with the UF ceramic (M3) providing the lowest overall irreversible fouling among all the membranes tested (5.1%).

Regarding the impact of chemical wash strength on recovery, increasing the NaOH concentration from 20 to 40 mM led to an increase in recovery from 2–4% to 4–6% for the polymeric membranes and from 4–7% to 7–12% for the ceramic membranes. For NaOCl, a lower concentration (250 mg Cl_2_/L) led to a flux recovery of 4–6% for polymeric membranes and 1–2% for ceramic membranes, whereas a higher concentration (500 mg Cl_2_/L) led to equivalent recoveries of 5–7% for all membranes. Overall, all the chemical washes provided low flux recoveries. According to these results, optimising physical cleaning appears to be a better strategy than using chemical washes to reduce operating costs, simplify operations, and minimise secondary pollution.

### 3.3. Mean Biofilm Thickness Formed on the Membrane Surface during Operation

**Figure 6:**
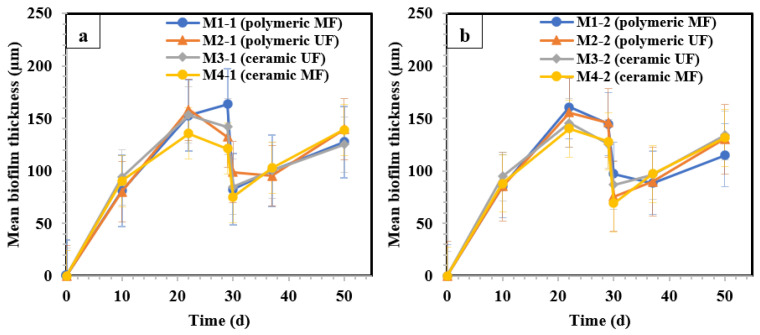
Evolution of mean biofilm thickness (µm) of (**a**) the membranes in Section 1 and (**b**) the membranes in Section 2. M1 (polymeric 0.1 µm MF), M2 (polymeric 0.03 µm UF), M3 (ceramic 300 kDa UF), and M4 (Lab-made ceramic MF). Day 30 is the physical and chemical cleaning day. The error bars show 95% confidence intervals.

To study the variation in the average thickness of the cake/biofilm accumulated on the membrane during filtration in Section 1 (Figure 6a) and Section 2 (Figure 6b), OCT imaging was performed on days 10, 22, 29, 30, 37, and 50. Raw OCT images of all the membranes before (day 29) and after cleaning (day 30) are shown in Figure S8. In Section 1, the mean cake/biofilm thickness of M1 (polymeric MF) and M2 (polymeric UF) was measured as 80 µm on day 10. For ceramic membranes of M3 (ceramic UF) and M4 (ceramic lab-made MF), the average thickness was 90 µm on the same day. The cake/biofilm thickness increased by more than 1.5 times for all the membranes until day 22. After that, the thicknesses experienced a slight decrease by approximately 15–20 µm for all the membranes except M2 (polymeric UF), which might be due to the detachment of the aged biofilm on the membrane surface as no cake compression was expected in the GDM process [32,33]. On day 30, after the cleaning procedure, the mean thickness decreased substantially to 82, 98, 84, and 75 µm for M1 (polymeric MF), M2 (polymeric UF), M3 (ceramic UF), and M4 (ceramic lab-made MF), respectively. Physical cleaning (increased hydraulic reversible fouling removal) explains the marked decrease in biofilm/cake thickness from the membrane surface. Subsequently, the thickness increased almost linearly by day 50 after the filtration. The mean biofilm thickness was not significantly different between Section 1 and Section 2 (*p* > 0.05) on day 30. After the membrane cleaning event on day 30 in Section 2, the thickness was 97, 86, 75, and 69 µm for M1 (polymeric MF), M2 (polymeric UF), M3 (ceramic UF), and M4 (ceramic MF), respectively. In Section 1, which was subjected to an enhanced air/water backwash, which resulted in higher flux recoveries (higher hydraulic reversible fouling removal), a lower mean biofilm thickness was expected for all membranes compared to those in Section 2. However, in practice, only two out of four membranes (polymeric MF and ceramic MF) showed lower thicknesses owing to more aggressive cleaning.

### 3.4. Permeate Quality of the Polymeric and Ceramic Membranes during the Operation

**Figure 7:**
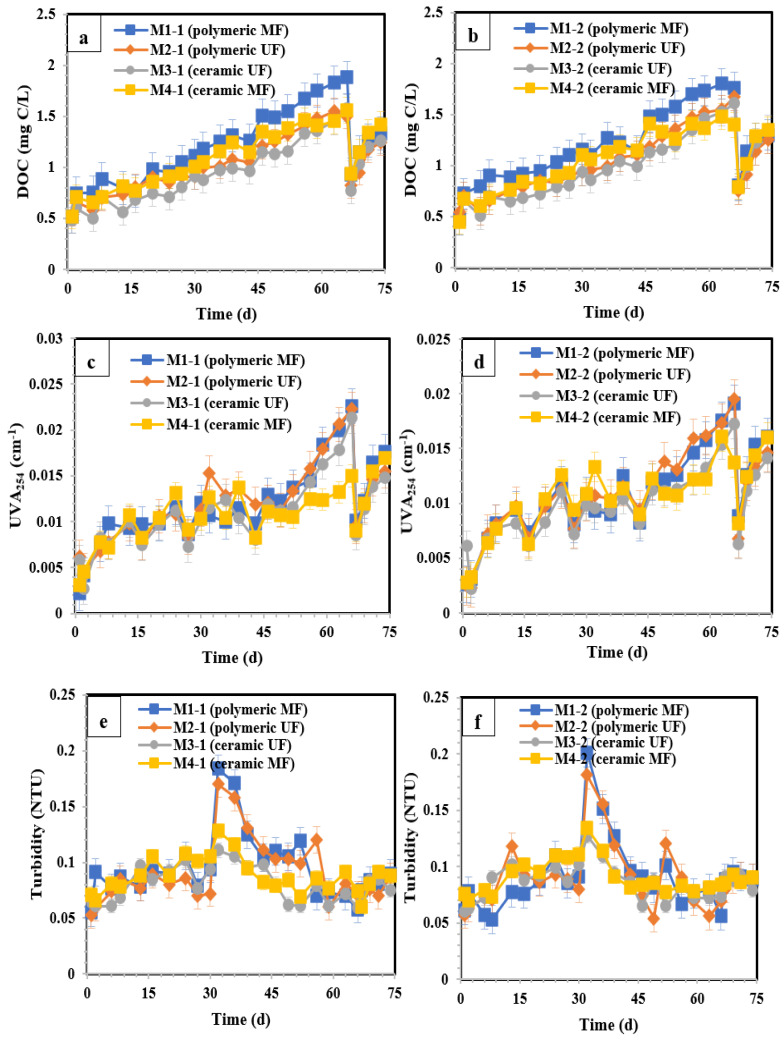
Variation in (**a**) DOC in Section 1, (**b**) DOC in Section 2, (**c**) UVA_254_ in Section 1, (**d**) UVA_254_ in Section 2, (**e**) turbidity in Section 1, and (**f**) turbidity in Section 2 during the filtration time. M1 (polymeric 0.1 µm MF), M2 (polymeric 0.03 µm UF), M3 (ceramic 300 kDa UF), and M4 (Lab-made ceramic MF). Days 30 and 68 are the membrane cleaning and resin regeneration days, respectively. The error bars show 95% confidence intervals.

The evolution of the permeate DOC of the membranes in Section 1 and Section 2 is shown in Figure 7a,b. In Section 1, the DOC concentrations of membrane effluents started at approximately 0.5 mg C/L, and rose progressively to 1.88, 1.55, 1.58, and 1.56 mg C/L for M1 (polymeric MF), M2 (polymeric UF), M3 (ceramic UF), and M4 (ceramic MF), respectively by day 68. M1 (polymeric MF) removed DOC by approximately 15%, whereas DOC removal using M2 (polymeric UF), M3 (ceramic UF), and M4 (ceramic MF) was approximately 30% on Day 68, compared to the DOC of the BIEX column effluents. Based on Figure S9a, the mean DOC of M3 (ceramic UF) from days 1 to 68 showed the lowest concentration (0.94 mg C/L) among all membranes. After resin regeneration on day 68, the permeate DOC of all the membranes decreased markedly to a value slightly higher than that on the initial day. The performance of the membranes for DOC removal in Section 2 shows a trend similar to that in Section 1. Likewise, according to Figure S9b, M3 (ceramic UF) showed the best performance of DOC removal (mean DOC during days 1–68 = 0.96 mg C/L) in Section 2. Therefore, it can be concluded that the increased air/water backwash flow and pressure (during physical cleaning) and chemical agent (NaOCl, NaOH) concentration (during chemical cleaning) in this study had no visible impact on DOC removal by membrane filtration.

UVA_254_ exhibited the same trend as DOC in both sections (Figure 7c,d). UVA_254_ in Section 1 started at approximately 0.003 cm^−1^ for all membranes. This figure then increased by a fluctuating trend to 0.023, 0.022, 0.021, and 0.015 cm^−1^ for M1 (polymeric MF), M2 (polymeric UF), M3 (ceramic UF), and M4 (ceramic MF), respectively, by day 68. UVA_254_ removal using M1 (polymeric MF), M2 (polymeric UF), and M3 (ceramic UF) on day 68 was approximately 37%, whereas M4 (ceramic MF) removed approximately 57% of the UVA_254_ compared to the effluent of the BIEX column. According to Figure S9c,d, the mean of UVA_254_ from days 1 to 68 was approximately 0.01 cm^−1^ for all the tested membranes in both sections, which is a very high performance. Similarly, no marked influence of variations in air/water backwash flow, pressure, and chemical agent concentration during physical and chemical cleaning on UVA_254_ removal was observed. As expected, resin regeneration on day 68 decreased UVA_254_ in permeates in both sections to approximately 0.009 cm^−1^. In Section 2, the performance of the membranes in terms of UVA_254_ removal showed a trend very similar to that in Section 1.

The evolution of turbidity during filtration in Section 1 and Section 2 is shown in Figure 7e and Figure 7f, respectively. In Section 1, for days 1–29 (before membrane cleaning), the polymeric (M1 and M2) and ceramic (M3 and M4) membranes showed similar performances. Turbidity started at approximately 0.05 NTU, followed by a fluctuating upper trend to 0.05–0.1 NTU until day 29. Immediately after membrane physical and chemical cleaning on day 30, turbidity experienced a peak at approximately 0.17 NTU for M1 (polymeric MF) and M2 (polymeric UF), and 0.12 NTU for M3 (ceramic UF) and M4 (ceramic MF). Attributing this to the higher mechanical strength of the ceramic membranes during physical cleaning, the turbidity peaked at lower values for these membranes after physical cleaning compared to the polymeric membranes. Then, the turbidity of all the membranes decreased and reached a stable value of approximately 0.09 NTU at the end of the filtration time. Based on Figure S9e,f, which was plotted using the turbidity data for the entire study, the membranes showed a turbidity of 0.08–0.09 NTU (the mean of the turbidity during the whole filtration time). Based on the turbidity results, the physical and chemical cleaning processes of the membrane increased permeate turbidity for only a short period. Finally, the enhanced cleaning conditions (in Section 1) had no marked impact on the turbidity of the membrane permeates compared to those in Section 2.

## 4. Conclusions

Two commercial polymeric membranes of 0.1 µm MF (M1) and 0.03 µm UF (M2) and two ceramic membranes (300 kDa commercial UF (M3) and lab-made MF (M4)) were used in the BIEX + GDM hybrid process for treating river water. The performance of both polymeric and ceramic membranes in terms of flux and permeate quality was studied. Furthermore, physical (air + water backwash) and chemical cleaning (NaOH 20 and 40 mM, NaOCl 250 and 500 mg Cl_2_/L) were performed to study the dominant fouling type causing the flux decrease in the membranes. Finally, the impacts of increasing the air/water backwash flow and pressure (during physical cleaning) and chemical cleaning agent concentration (during chemical cleaning) on stabilised flux, flux recovery, and permeate quality were investigated. The most important results obtained were as follows:

- The BIEX resin column successfully reduced DOC from 7.04 mg C/L to below 2 mg C/L over the 68 days of operation.
- Before membrane cleaning (days 1–29), the fluxes of the membranes stabilised after day 8 at approximately 4.5–5 LMH. After membrane cleaning (days 30–73), the flux was restabilised after 24 days (i.e., on day 54). The new stabilised flux of the membranes was approximately 3.7–5.5 LMH at the end of filtration, which was statistically significant. However, this difference was not substantial, indicating that the membrane material is not a key factor.
- Before cleaning, the ceramic and polymeric membranes showed similar stabilised fluxes. After the cleaning process, the stabilised flux of the ceramic membranes decreased or remained constant, whereas that of the polymeric membranes increased under both enhanced and decreased cleaning conditions compared to that before cleaning.
- The majority of membrane fouling (57–80%) was hydraulically reversible. The membranes in Section 1, with enhanced air/water backwash flow and pressure, showed higher hydraulic reversible fouling removal than those in Section 2. Hydraulically reversible fouling removal was more efficient with ceramic membranes than with polymeric membranes. Chemical cleaning was not effective for removing hydraulically irreversible foulants. Therefore, we recommend performing physical learning and eliminating chemical cleaning steps.
- The ceramic UF (M3) membrane showed the best permeate quality, with DOC and UVA_254_ removals of 30% and 37% on day 68, respectively, as measured against the influent (i.e., the BIEX effluent).
- Physical and chemical cleaning had no measurable impact on DOC and UVA_254_, whereas physical cleaning increased the turbidity of the membranes for a short period. Resin regeneration decreased the DOC and UVA_254_ of the membrane permeate, whereas it had no significant impact on membrane turbidity.

According to the obtained results, most of the membrane fouling in the hybrid BIEX + GDM process was hydraulically reversible using an air/water backwash. Therefore, future research should focus on optimising physical cleaning to increase flux recovery and stabilise flux.

## Figures and Tables

**Table 1:** Characteristics of the membranes used in the filtration section.

Membrane Name	Membrane Type	Pore Size	Membrane Material	Membrane Area (cm^2^)	Suppliers
M1-1 * M1-2	Flat-sheet disk-shaped Polymeric	0.1 µm (MF)	Polyether Sulfone (PES)	17	Sterlitech, Auburn, WA, USA
M2-1 M2-2	Flat-sheet disk-shaped Polymeric	0.03 µm (UF)	PES	17	Sterlitech, Auburn, WA, USA
M3-1 M3-2	Flat-sheet disk-shaped Ceramic	300 kDa (UF)	ZrO_2_/TiO_2_	17	Tami Industries, Nyons, France
M4-1 M4-2	Flat-sheet disk-shaped Ceramic	0.62 ± 0.06 µm ** (MF)	Kaolin support + alumina layer	15 ± 0.09 **	Lab-made

* The use of -1 and -2 refer to Section 1 (pilot 1) and Section 2 (pilot 2) in Figure 1, respectively. For example, M1-1 represents M1 in Section 1 (pilot 1), and M1-2 represents M1 in Section 2 (pilot 2). ** Mean ± 95% confidence interval.

**Table 2:** Characteristics of the influent water from the Des Prairies River.

Parameters	Mean ± 95% Confidence Interval	Number of Samples
DOC (mg C/L)	7.04 ± 0.18	27
Turbidity (NTU)	5.09 ± 0.27	27
pH	7.17 ± 0.04	27
Alkalinity (mg CaCO_3_/L)	23.8 ± 0.4	27
UVA_254_ (cm^−1^)	0.21 ± 0.005	27
Nitrate (mg/L)	2.71 ± 0.11	23
Sulphate (mg/L)	3.57 ± 0.37	23
Chloride (mg/L)	5.04 ± 0.15	23

**Table 3:** Stepwise description of physical and chemical membrane cleaning.

No.	Step Name	Descriptions	Figure
1	Turning the membranes face down	Membrane positions were faced down according to the inlet flow	Figure S4a
2	Backwash with air	(Section 1) P = 30 psi, Q = 5 L/h, t = 2 min (Section 2) P = 15 psi, Q = 2.5 L/h, t = 2 min	Figure S4b,c
3	Backwash with DI water	(Section 1) Water head = 120 cm, t = 4 h (Section 2) Water head = 90 cm, t = 4 h	Figure S4d
4	Returning the membranes face up	Membrane positions were returned to the normal filtration position.	Figure S4e
5	DI water flux measurement	Measuring DI water flux at a water head of 90 cm for 15 min in both sections	Figure S4f
6	Chemical cleaning using NaOH	(Section 1) NaOH = 40 mM, Water head = 90 cm, t = 6 h (Section 2) NaOH = 20 mM, Water head = 90 cm, t = 6 h	Figure S4g
7	DI water flux measurement	Measuring DI water flux at the water head of 90 cm for 15 min in both sections	Figure S4h
8	Chemical cleaning using NaOCl	(Section 1) NaOCl = 500 mg Cl_2_/L, Water head = 90 cm, t = 6 h (Section 2) NaOCl = 250 mg Cl_2_/L, Water head = 90 cm, t = 6 h	Figure S4i
9	DI water flux measurement	Measuring DI water flux at the water head of 90 cm for 15 min in both sections	Figure S4j

**Table 4:** Mean *±* 95% confidence interval of the membrane’s stabilised flux in both sections before and after membrane cleaning. Before cleaning from day 8 to 29; after cleaning from day 54 to 73.

Membrane Type		Section 1 (Enhanced Cleaning)				Section 2 (Decreased Cleaning)			
		Polymeric		Ceramic		Polymeric		Ceramic	
		MF (M1-1)	UF (M2-1)	UF (M3-1)	MF (M4-1)	MF (M1-2)	UF (M2-2)	UF (M3-2)	MF (M4-2)
Stabilised flux (LMH)	Before cleaning (day 8–29)	4.46 ± 0.42	4.54 ± 0.31	4.56 ± 0.22	4.50 ± 0.27	4.77 ± 0.37	4.46 ± 0.51	5.04 ± 0.23	4.50 ± 0.17
	After cleaning (day 54–73)	5.13 ± 0.14	4.69 ± 0.06	4.67 ± 0.14	4.00 ± 0.11	5.34 ± 0.11	5.41 ± 0.11	4.61 ± 0.25	3.75 ± 0.12
	Change (%)	+15%	+3.3%	+2.8%	−11%	+12%	+21%	−8.5%	−16%

**Table 5:** Flux recovery percentages obtained via physical and chemical cleaning (mean ± 95% confidence interval).

Membrane Type		Section 1 (Enhanced Cleaning)				Section 2 (Decreased Cleaning)			
		Polymeric		Ceramic		Polymeric		Ceramic	
		MF (M1-1)	UF (M2-1)	UF (M3-1)	MF (M4-1)	MF (M1-2)	UF (M2-2)	UF (M3-2)	MF (M4-2)
Flux recovery %	Physical cleaning (air + water)	67.3 ± 8.2	69.7 ± 3.3	79.1 ± 6.0	79.6 ± 4.4	57.0 ± 2.1	61.3 ± 5.3	75.5 ± 10.5	70.8 ± 8.0
	Chemical cleaning with NaOH	4.9 ± 4.5	6.1 ± 4.5	12.8 ± 3.2	7.8± 4.1	2.5 ± 2.1	3.8 ± 2.8	6.7 ± 6.4	4.6 ± 3.9
	Chemical cleaning with NaOCl	7.5 ± 3.9	4.9 ± 2.7	5.3 ± 1.6	5.1 ± 4.0	6.2 ± 4.2	4.4 ± 2.7	3.0± 2.0	1.1 ± 0.5

**Table 6:** Percentages of different fouling types causing a flux decline in the filtration.

Membrane Type		Section 1 (Enhanced Cleaning)				Section 2 (Decreased Cleaning)			
		Polymeric		Ceramic		Polymeric		Ceramic	
		MF (M1-1)	UF (M2-1)	UF (M3-1)	MF (M4-1)	MF (M1-2)	UF (M2-2)	UF (M3-2)	MF (M4-2)
Different fouling types %	Hydraulically reversible (%)	67.3	69.7	79.1	79.6	57.0	61.3	75.5	70.8
	Hydraulically irreversible (%)	32.7	30.3	20.9	20.4	43.0	38.7	24.5	29.2
	Chemically reversible (%)	12.4	11.0	18.1	12.9	8.7	8.2	9.7	5.7
	Chemically irreversible (%)	20.3	19.3	2.8	7.5	34.3	30.5	14.8	23.5

## Data Availability

The data are contained in this article.

## Supplementary Materials

The following supporting information can be downloaded at: https://www.mdpi.com/article/10.3390/membranes14020033/s1, Figure S1. Schematic of M4 (lab-made) production steps. Figure S2. SEM micrograph of the Lab-made membrane, demonstrating the kaolin support and the top alumina layer. Figure S3: (a) SEM micrograph of the Lab-made membrane. Spectrums 1 and 2 show a small area in the kaolin support and alumina top layer. (b) The Energy Dispersive X-ray (EDX) spectra of point 1 (in kaolin support), and c) EDX spectra of point 2 (in the top alumina layer). Figure S4: Schematic of the steps in physical and chemical cleaning of the membranes. Figure S5: Dynamics of cumulative anion exchange on the resin of BIEX column 2 during the operation. Figure S6: Variations of (a) UVA_254_ and (b) turbidity of BIEX column effluent during the operation. Figure S7: (a) Variation in fouling resistance of membranes in Section 1 during the filtration, and (b) Variation in fouling resistance of membranes in Section 2 during the filtration. Figure S8: OCT images of (a) M1-1 (polymeric MF in Section 1) before cleaning, (b) M1-1 (polymeric MF in Section 2) after cleaning, (c) M2-1 (polymeric UF in Section 1) before cleaning, (d) M2-1 (polymeric UF in Section 1) after cleaning, (e) M3-1 (ceramic UF in Section 1) before cleaning, (f) M3-1 (ceramic UF in Section 1) after cleaning, (g) M4-1 (ceramic MF in Section 1) before cleaning, (h) M4-1 (ceramic MF in Section 1) after cleaning, (i) M1-2 (polymeric MF in Section 2) before cleaning, (j) M1-2 (polymeric MF in Section 2) after cleaning, (k) M2-2 (polymeric UF in Section 2) before cleaning, (l) M2-2 (polymeric UF in Section 2) after cleaning, (m) M3-2 (ceramic UF in Section 2) before cleaning, (n) M3-2 (ceramic UF in Section 2) after cleaning, (o) M4-2 (ceramic MF in Section 2) before cleaning, and (p) M4-2 (ceramic MF in Section 2) after cleaning. Figure S9: (a,b) Mean DOC in Section 1 and Section 2 days 1–68; (c,d) Mean UVA_254_ in Section 1 and Section 2 days 1–68, (e,f) Mean turbidity in Section 1 and Section 2 during the whole operation period. M1 (polymeric 0.1 µm MF), M2 (polymeric 0.03 µm UF), M3 (ceramic 300 kDa UF), and M4 (Lab-made ceramic MF). The error bars show 95% confidence intervals. Table S1: Porosity, mean pore size, and water flux of the lab-made membranes. Mean ± 95% confidence interval.

## Abbreviations

The following abbreviations are used in this manuscript:

ANOVA	Analysis of variance
BIEX	Biological ion exchange
BV	Bed volume
DI water	Deionised water
DOC	Dissolved organic carbon
EBCT	Empty bed contact time
EDX	Energy Dispersive X-ray
GDM	Gravity-driven membrane
IEX	Ion exchange
LMH	L/m^2^h
MF	Microfiltration
MWCO	Molecular weight cut-off
NOM	Natural organic matter
OCT	Optical coherence tomography
PES	Polyethersulfone
PVC	Polyvinyl chloride
PVDF	Polyvinylidene difluoride
SEM	Scanning electron microscopy
SiC	Silicon carbide
TOC	Total organic carbon
UF	Ultrafiltration
UVA254	UV-absorbance at 254 nm wavelength
