# Supplementary material for: Impact of Cleaning on Membrane Performance during Surface Water Treatment: A Hybrid Process with Biological Ion Exchange and Gravity-Driven Membranes
Source: Membranes (Basel). 2024 Jan 25;14(2):33. doi: 10.3390/membranes14020033 (PMC10890121; doi:10.3390/membranes14020033)
Supplement: Supplementary file 1 [file membranes-14-00033-s001.zip › membranes-2814612-supplementary.pdf]

Supplementary Information

Impact of Cleaning on Membrane Performance during Surface Water Treatment: A hybrid Process with Biological Ion Exchange and Gravity-Driven Membranes

Yaser Rasouli [1], Benoit Barbeau [1], Raphaël Maltais-Tariant [2], Caroline Boudoux [2, 3], Dominique Claveau-Mallet [a, *]

[1] Department of Civil, Geological & Mining Engineering, Polytechnique Montréal, 2900 Boulevard Édouard-Montpetit, Montréal, Québec, H3T 1J4, Canada

[2] Department of Engineering Physics, Polytechnique Montréal, 2900 Édouard-Montpetit, Montréal, Québec, H3T 1J4, Canada

[3] Castor Optics, Inc., St-Laurent, Québec, Canada

[*]Corresponding author. Email: dominique.claveau-mallet@polymtl.ca

Membrane characterizations

The determination of membrane porosity involved the calculation based on the amount of water absorbed by the membrane structure following immersion in a water bath. Initially, the dry membranes, denoted as "W_1," were weighed. Subsequently, these membranes were immersed in pure water at a consistent ambient temperature for a duration of 72 hours. Following this soaking period, the outer surface of the membranes was dried using Kimwipes™ and subsequently reweighed as "W_2." The formula employed to compute membrane porosity was in accordance with the method outlined by (Arzani, Mahdavi et al. 2016):

$$Porosity = \left(\frac{W_1 - W_2}{\rho_m V_m}\right)$$

where ρ_m and V_m are the density of pure water at the corresponding temperature and membrane volume, respectively.

The membrane mean pore radius (R_m) was calculated using the Guerout-Elford-Ferry equation (Zhang, Lang et al. 2015), where ε is the membrane porosity, L is the membrane thickness, μ

is the water viscosity at the filtration temperature, J is the membrane flux, and ΔP is the pressure.

$$R_m = \sqrt{\frac{(2.9-1.75\,\epsilon)8\mu\,L\,J}{\varepsilon\,\Delta P}}$$

Membrane structure characterization using scanning electron microscopy

A small part of the clean membranes was carefully cut and subjected to SEM analyses. The samples were prepared by vacuum coating with a very thin layer of gold (Polaron SC502 sputter coater) at a pressure of approximately 10 bar and current of 10 mA. The samples were observed on a device (Jeol, JSM-7600TFE, JEOL Ltd., Japan) using low electron voltages (5–10 kV).

Characterization results:

Table S1. Porosity, mean pore size and water flux of the lab-made membranes. Mean ± 95% confidence interval.

Membrane type	Porosity (%)	Mean pore size (µm)	Water flux at 90 mbar (LMH)
M4 (Ceramic lab-made MF)	35.17 ± 3.55	0.62 ± 0.06	108.04 ± 4.01

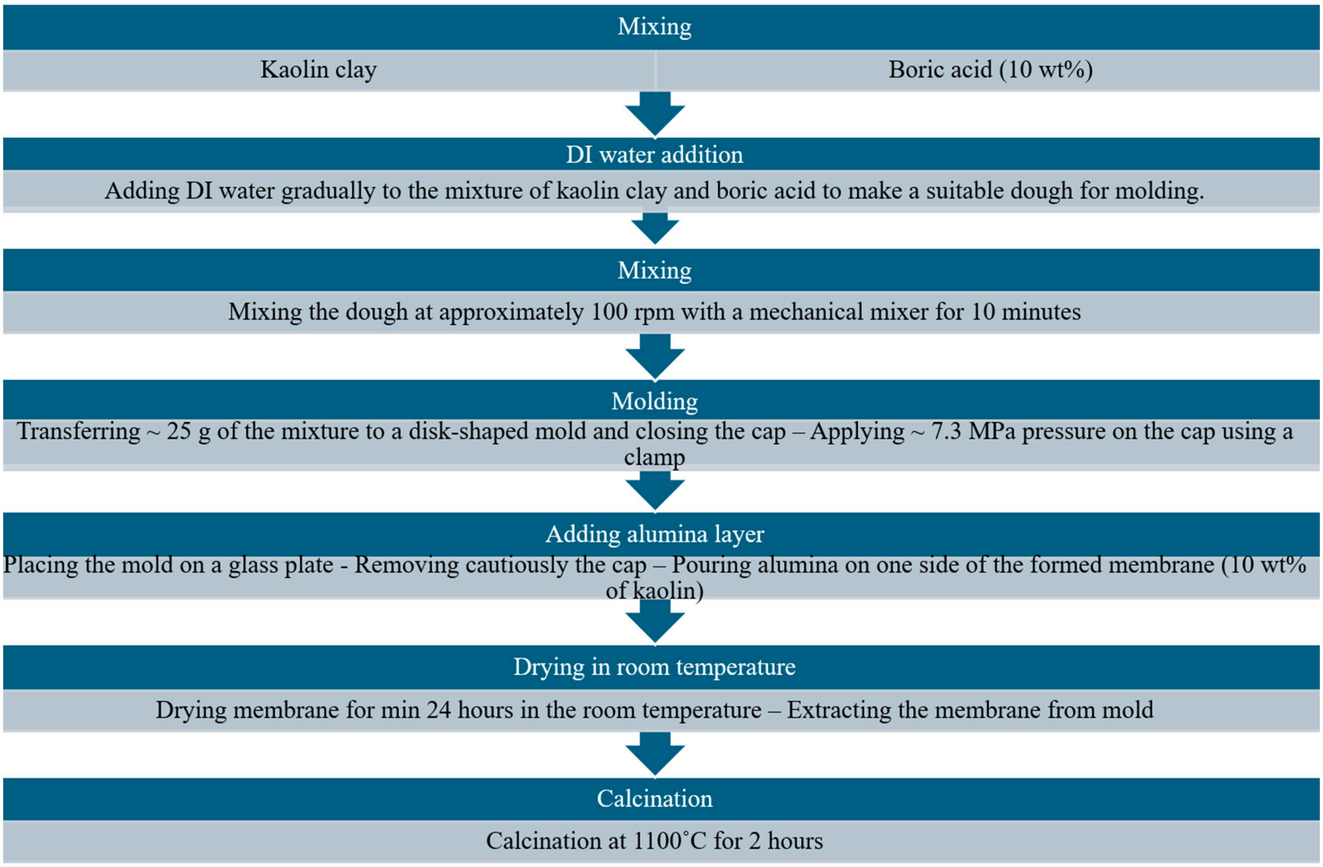

Figure S1. Schematic of M4 (lab-made) production steps

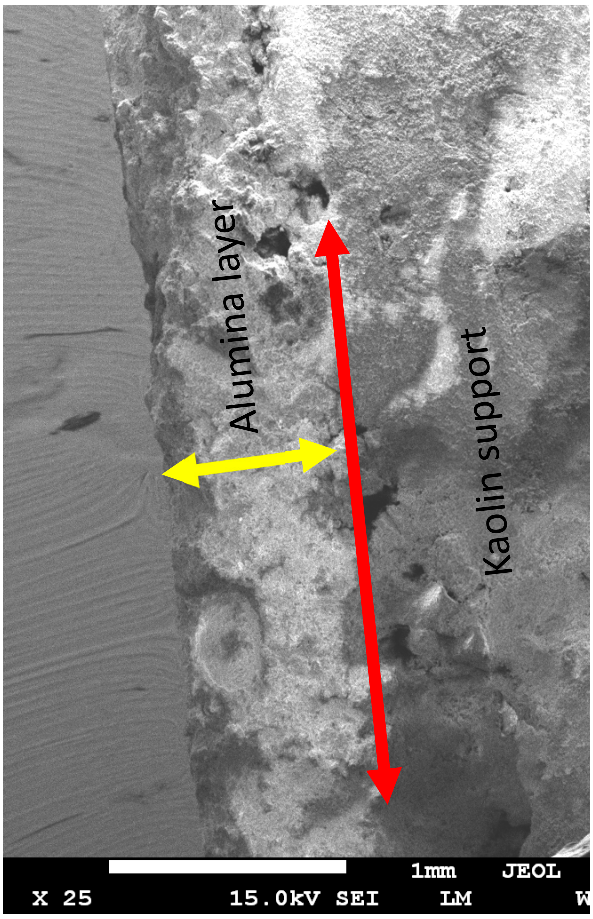

Figure S2. SEM micrograph of the Lab-made membrane, demonstrating the kaolin support and the top alumina layer

According to figure S3, point 1 which is in the kaolin support, shows more Si and less Al than Point 2 which is located in the alumina layer.

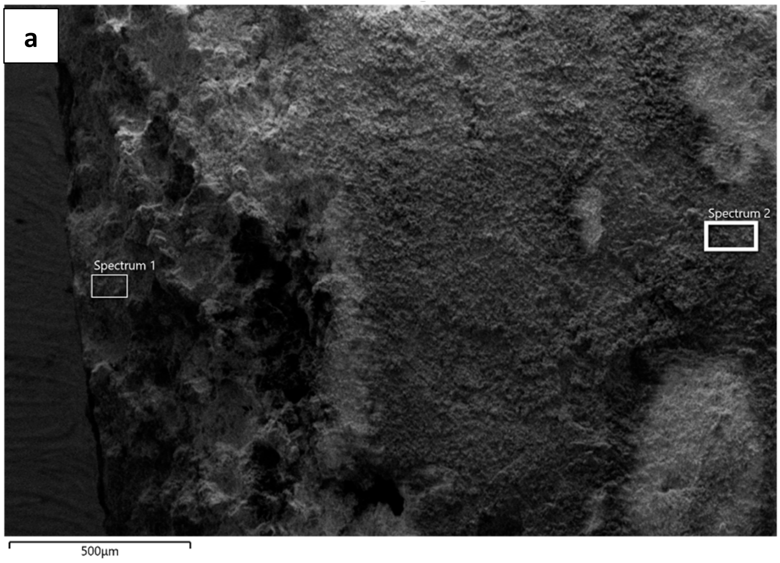

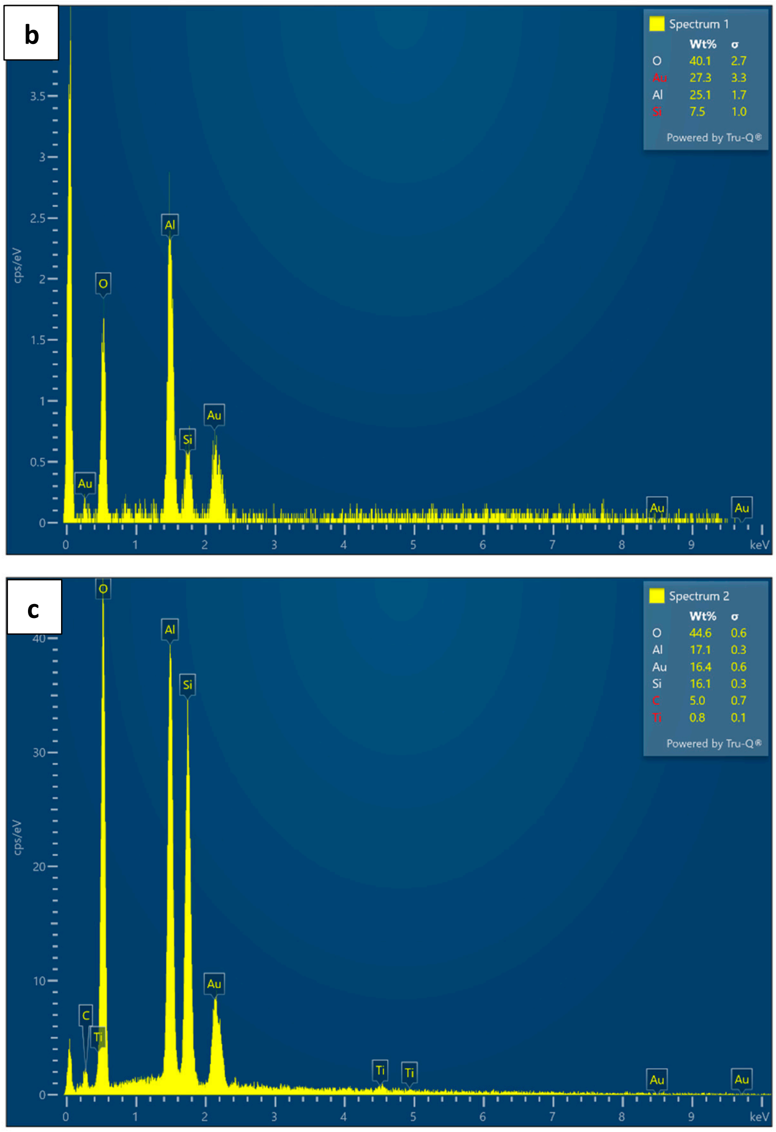

Figure S3. a) SEM micrograph of the Lab-made membrane. Spectrums 1 and 2 show a small area in the kaolin support and alumina top layer. b) The Energy Dispersive X-ray (EDX) spectra of point 1 (in kaolin support), and c) EDX spectra of point 2 (in top alumina layer).

Step 1: Turning the membranes face down – Membranes position was turned to face down according to the inlet flow

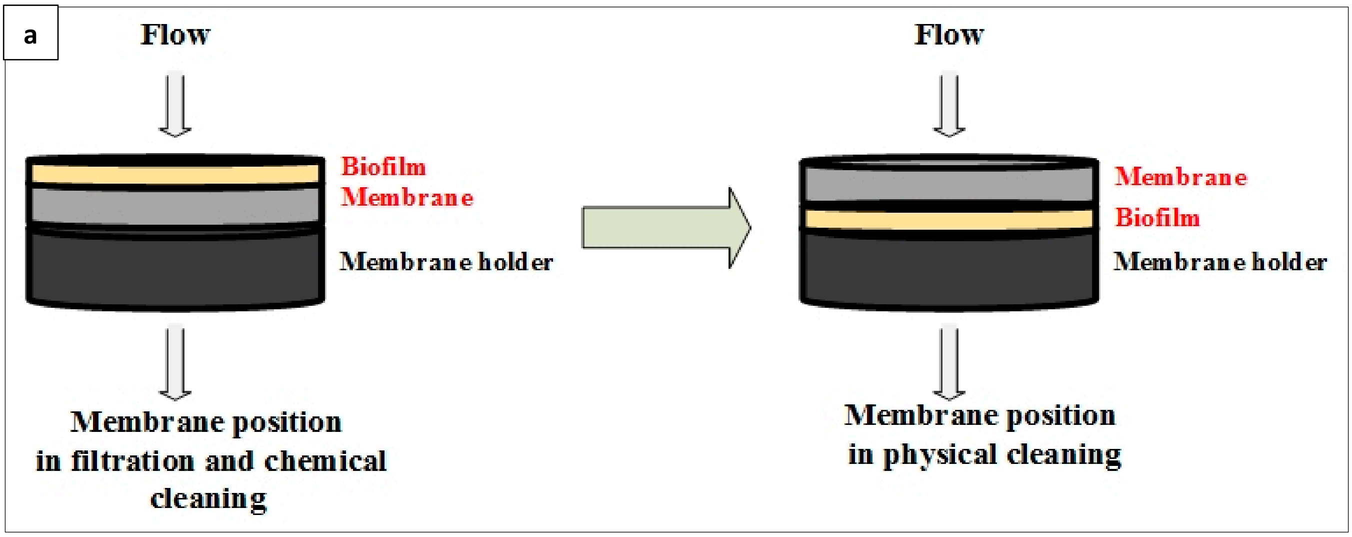

Step 2: Backwash with air at section 1) P = 30 psi, Q = 5 L/h, t = 2 min; section 2) P = 15 psi, Q = 2.5 L/h, t = 2 min.

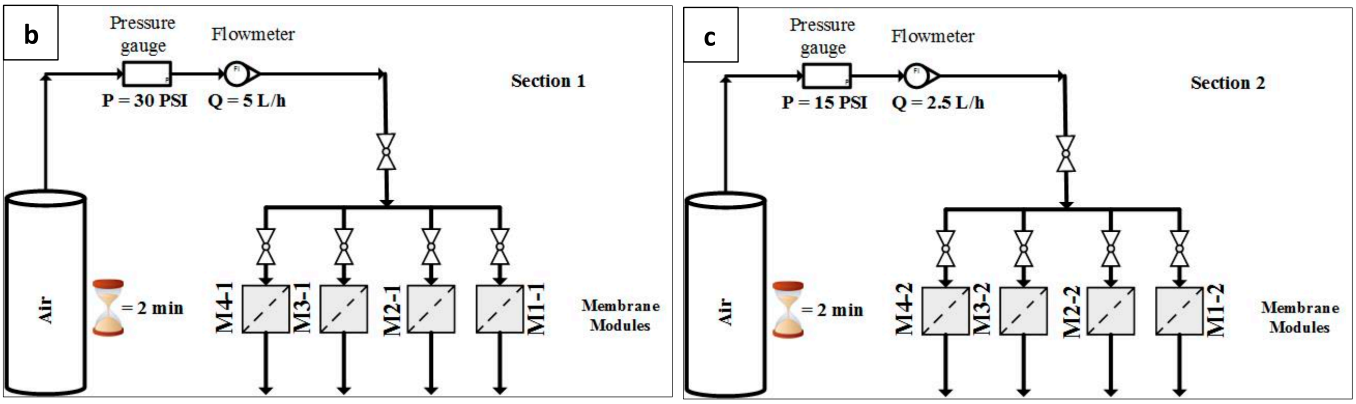

Step 3: Backwash with DI water at section 1) water head = 120 cm, t = 4 h; section 2) water head = 90 cm, t = 4 h.

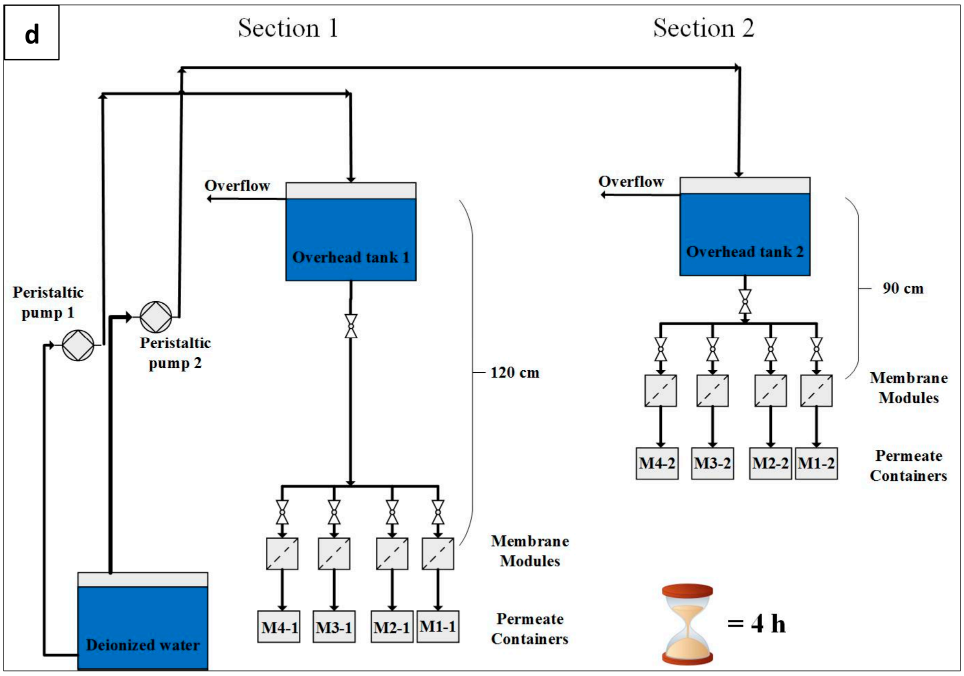

Step 4: Returning the membranes to normal position – Membranes position was turned face up again according to the inlet flow.

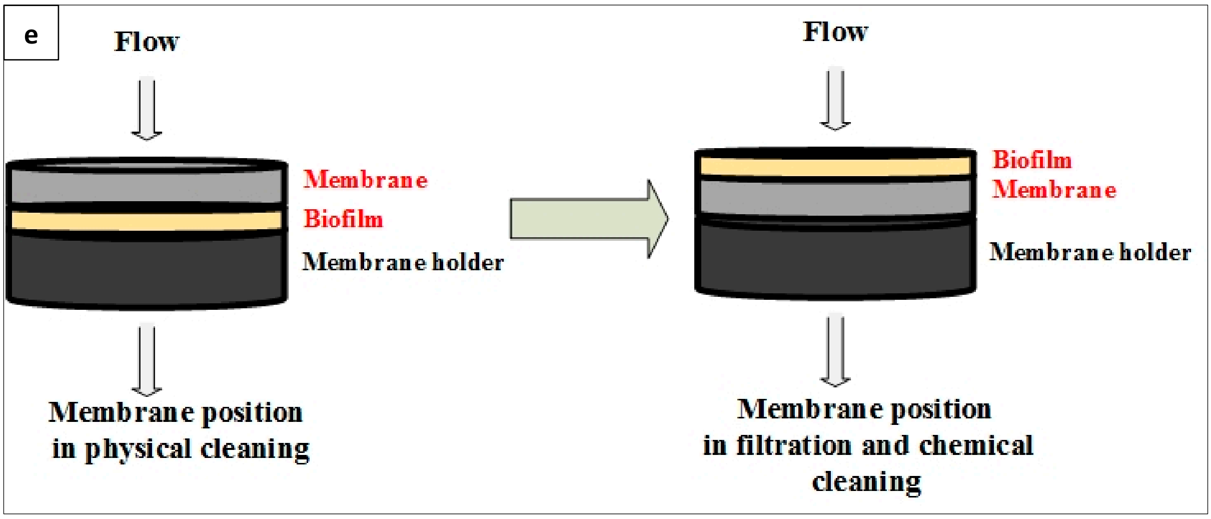

Step 5: Measuring DI water flux at water head of 90 cm for 15 min in both sections.

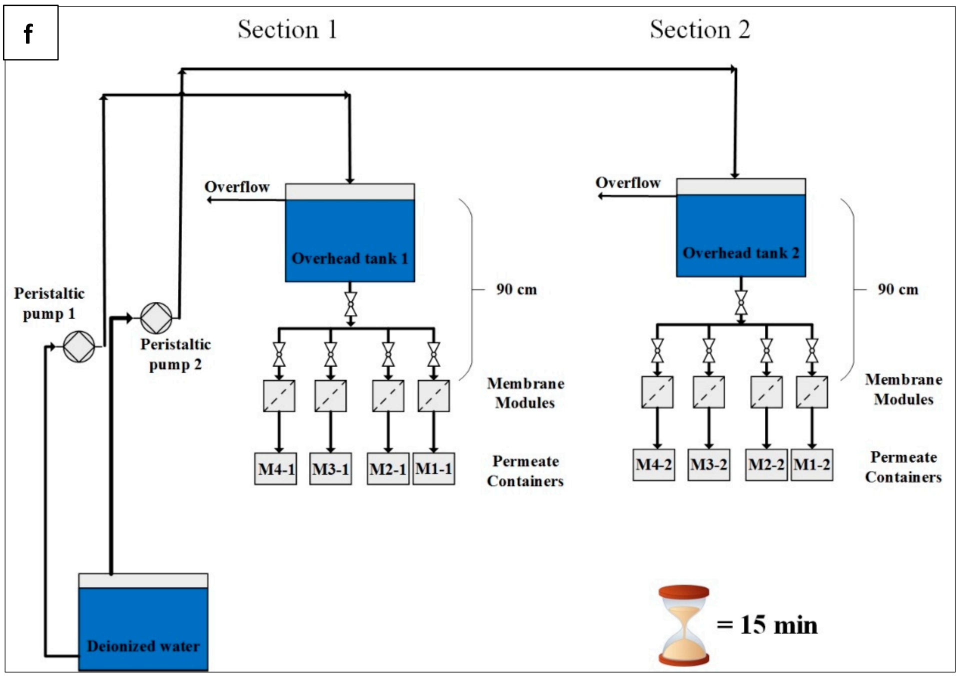

Step 6: Chemical cleaning by NaOH 40 mM in section 1 and 20 mM in section 2 both for 6 h at 90 cm water head.

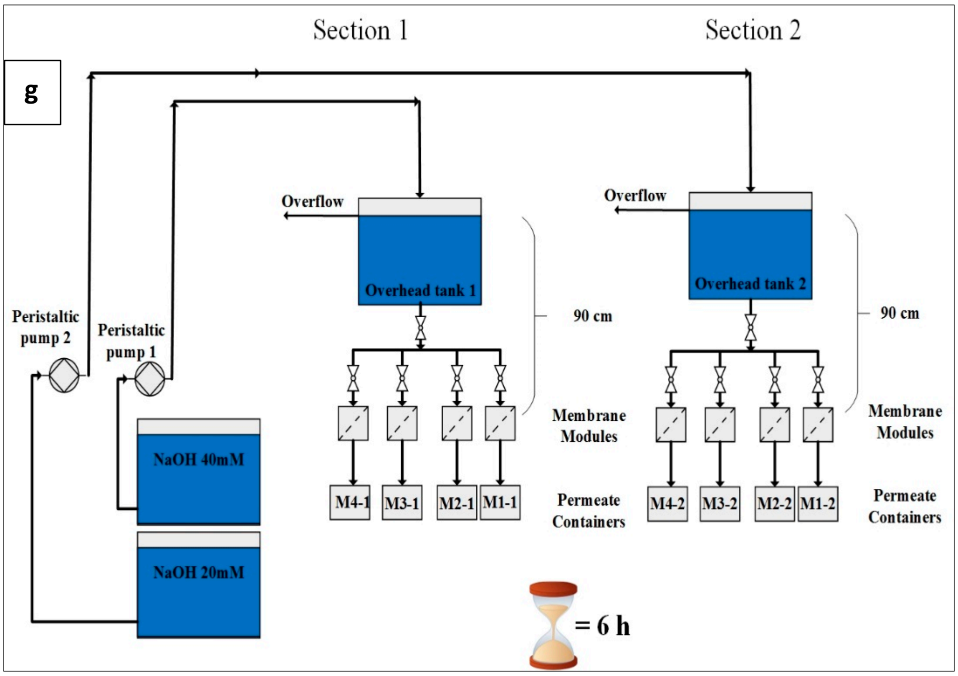

Step 7: Measuring DI water flux at water head of 90 cm for 15 min in both sections.

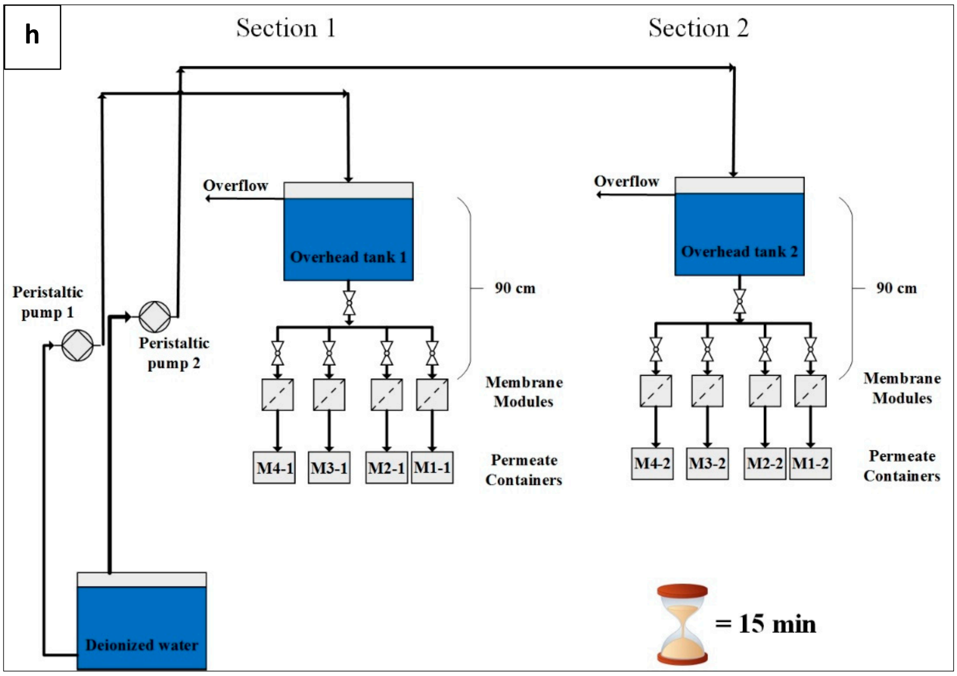

Step 8: Chemical cleaning by NaOCl 500 mg Cl2/L in section 1 and 250 mg Cl2/L in section 2 both at 90 cm water head for 6h.

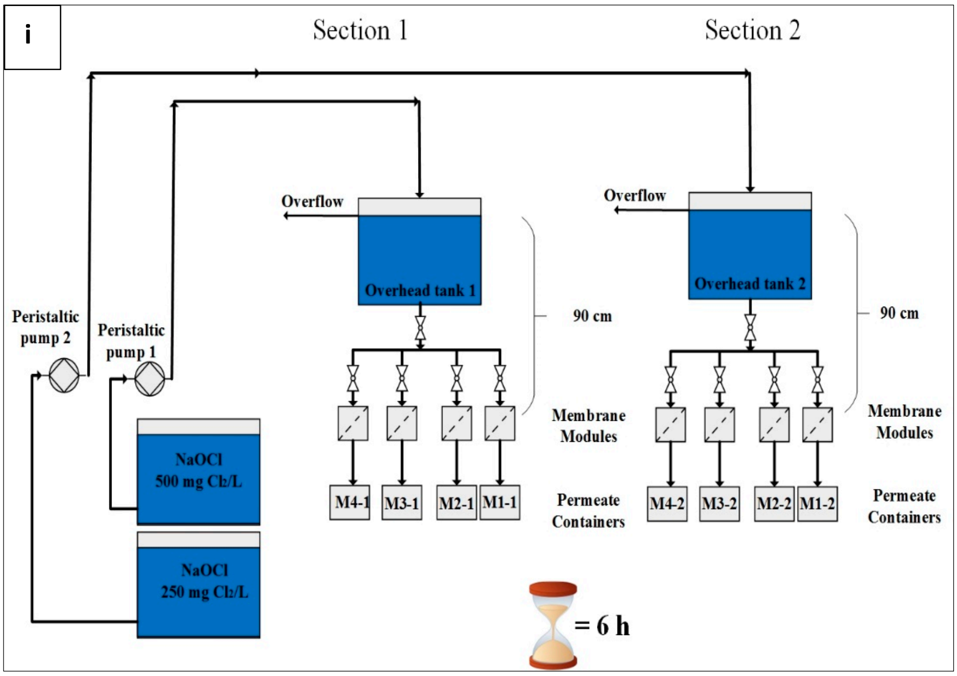

Step 9: Measuring DI water flux at water head of 90 cm for 15 min in both sections.

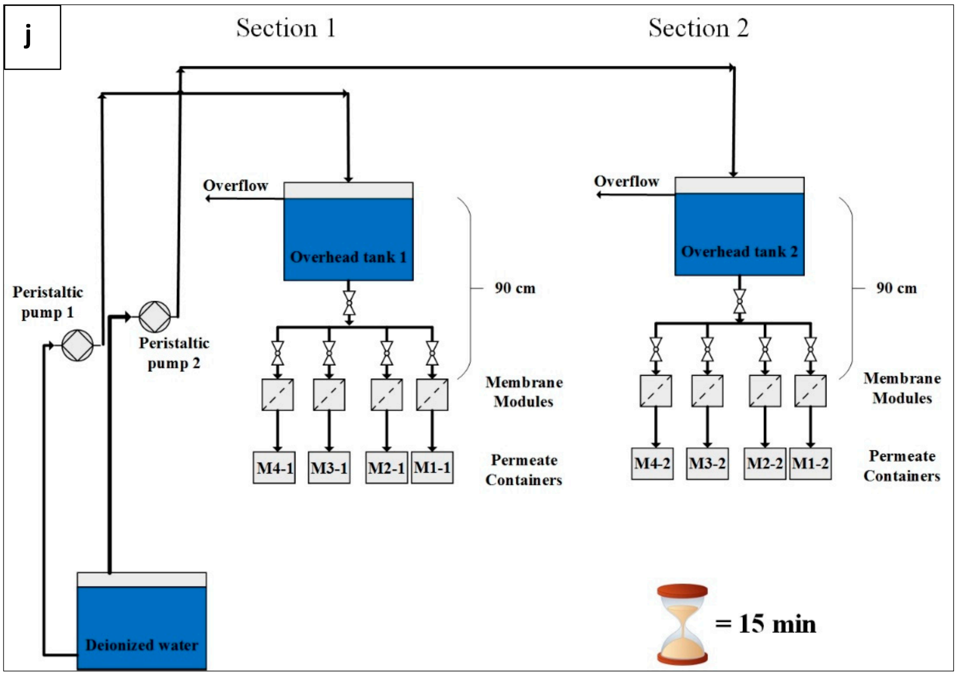

Figure S4. Schematic of the steps in physical and chemical cleaning of the membranes

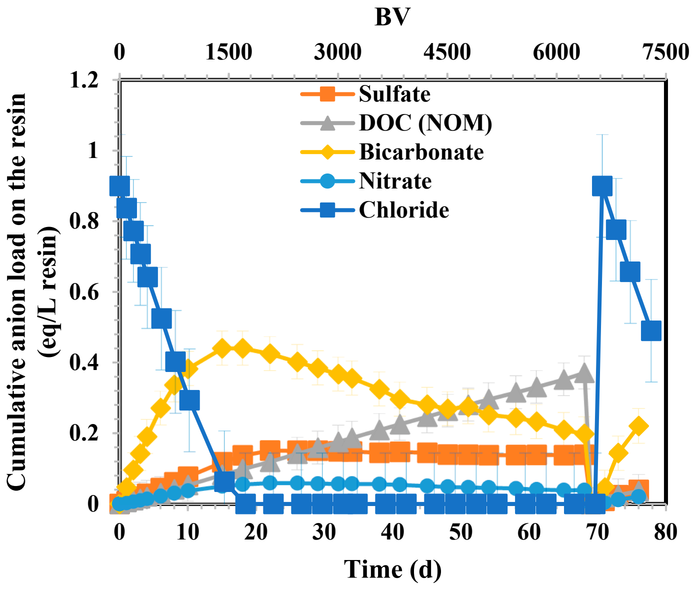

Figure S5. Dynamics of cumulative anion exchange on the resin of BIEX column 2 during the operation. Day 68 and 6,528 BV is the resin regeneration time. The error bars show 95% confidence intervals.

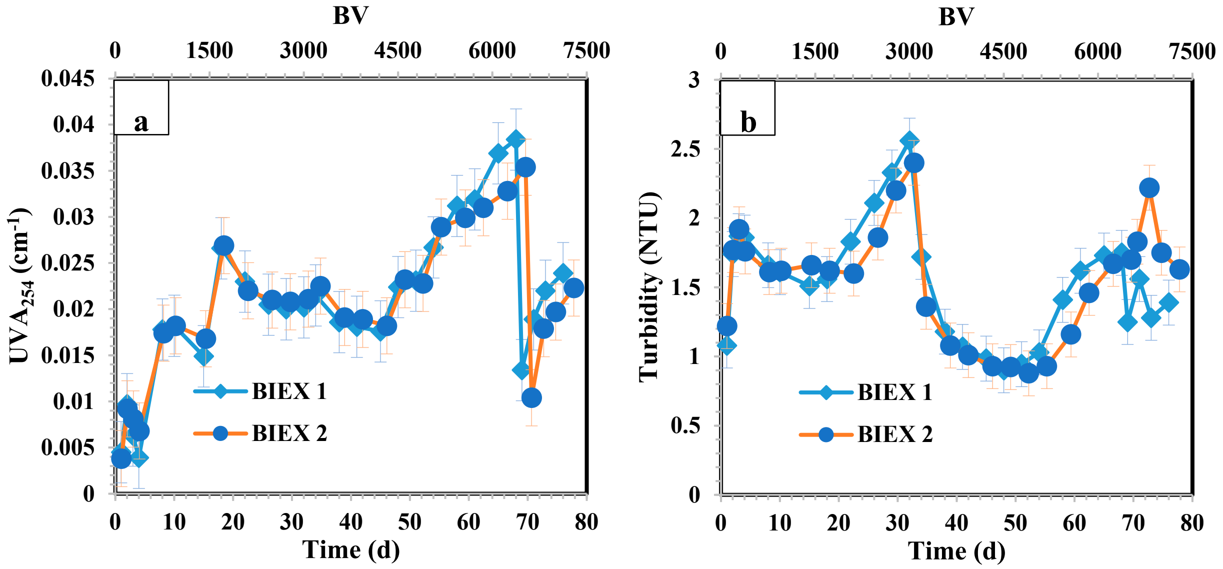

Figure S6. Variations of a) UVA_{254} and b) turbidity of BIEX column effluent during the operation. The error bars show 95% confidence intervals.

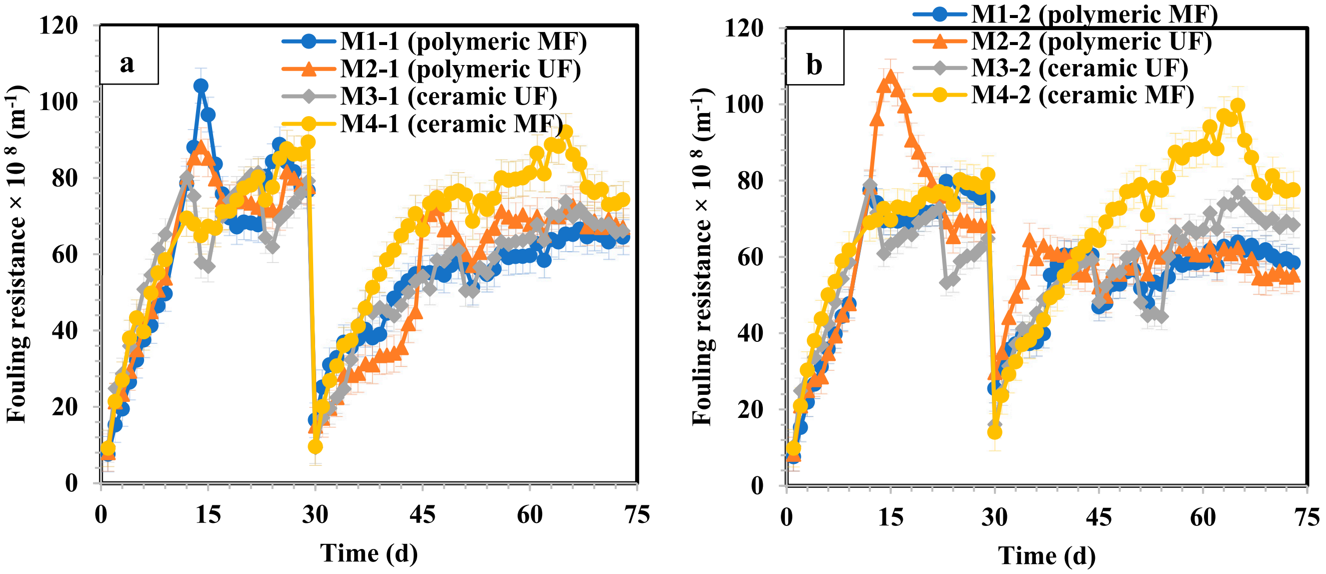

Figure S7. a) Variation of fouling resistance of membranes in section 1 during the filtration, and b) Variation of fouling resistance of membranes in section 2 during the filtration. M1 (polymeric 0.1 µm), M2 (polymeric 0.03 µm), M3 (ceramic 300 kDa), and M4 (Lab-made ceramic). Day 30 is the physical and chemical cleaning day. The error bars show 95% confidence intervals.

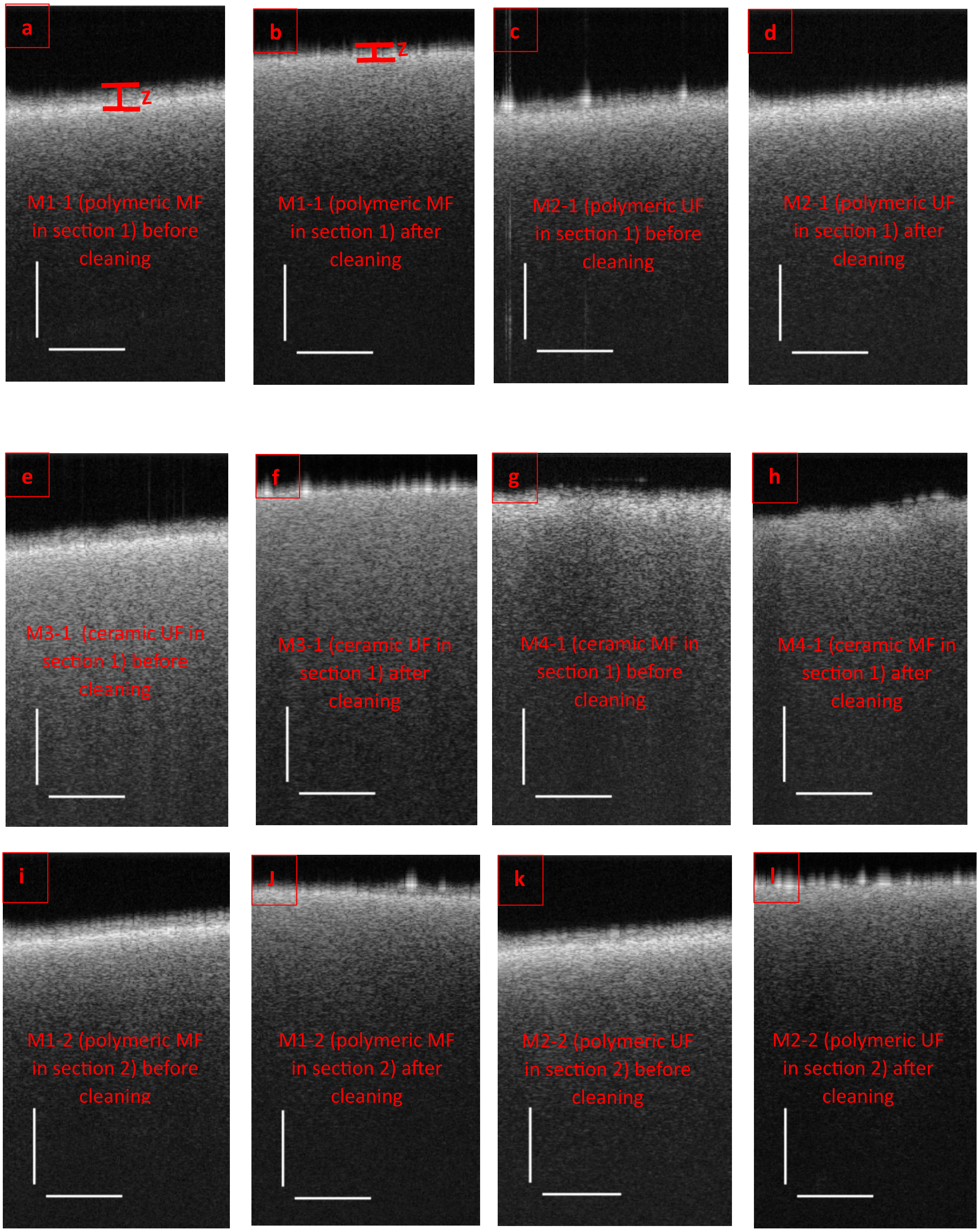
a
z
M1-1 (polymeric MF in section 1) before cleaning
b
z
M1-1 (polymeric MF in section 1) after cleaning
c
M2-1 (polymeric UF in section 1) before cleaning
d
M2-1 (polymeric UF in section 1) after cleaning
e
M3-1 (ceramic UF in section 1) before cleaning
f
M3-1 (ceramic UF in section 1) after cleaning
g
M4-1 (ceramic MF in section 1) before cleaning
h
M4-1 (ceramic MF in section 1) after cleaning
i
M1-2 (polymeric MF in section 2) before cleaning
J
M1-2 (polymeric MF in section 2) after cleaning
k
M2-2 (polymeric UF in section 2) before cleaning
l
M2-2 (polymeric UF in section 2) after cleaning

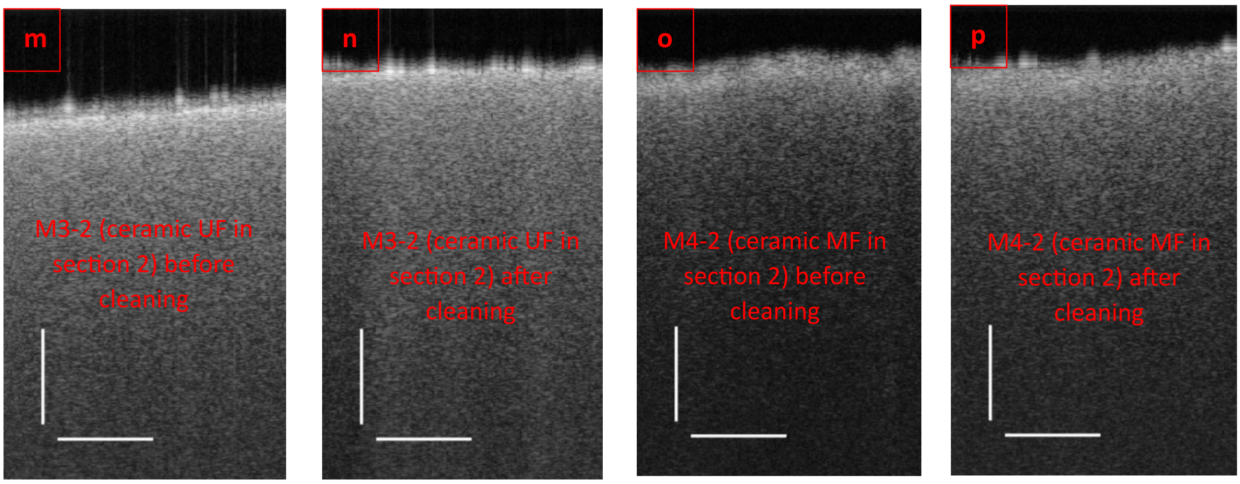

Figure S8. OCT images of a) M1-1 (polymeric MF in section 1) before cleaning, b) M1-1 (polymeric MF in section 2) after cleaning, c) M2-1 (polymeric UF in section 1) before cleaning, d) M2-1 (polymeric UF in section 1) after cleaning, e) M3-1 (ceramic UF in section 1) before cleaning, f) M3-1 (ceramic UF in section 1) after cleaning, g) M4-1 (ceramic MF in section 1) before cleaning, h) M4-1 (ceramic MF in section 1) after cleaning, i) M1-2 (polymeric MF in section 2) before cleaning, j) M1-2 (polymeric MF in section 2) after cleaning, k) M2-2 (polymeric UF in section 2) before cleaning, l) M2-2 (polymeric UF in section 2) after cleaning, m) M3-2 (ceramic UF in section 2) before cleaning, n) M3-2 (ceramic UF in section 2) after cleaning, o) M4-2 (ceramic MF in section 2) before cleaning, and p) M4-2 (ceramic MF in section 2) after cleaning. M1 (polymeric 0.1 µm MF), M2 (polymeric 0.03 µm UF), M3 (ceramic 300 kDa UF), and M4 (Lab-made ceramic MF). X and y-axis bars show 1 mm distance.

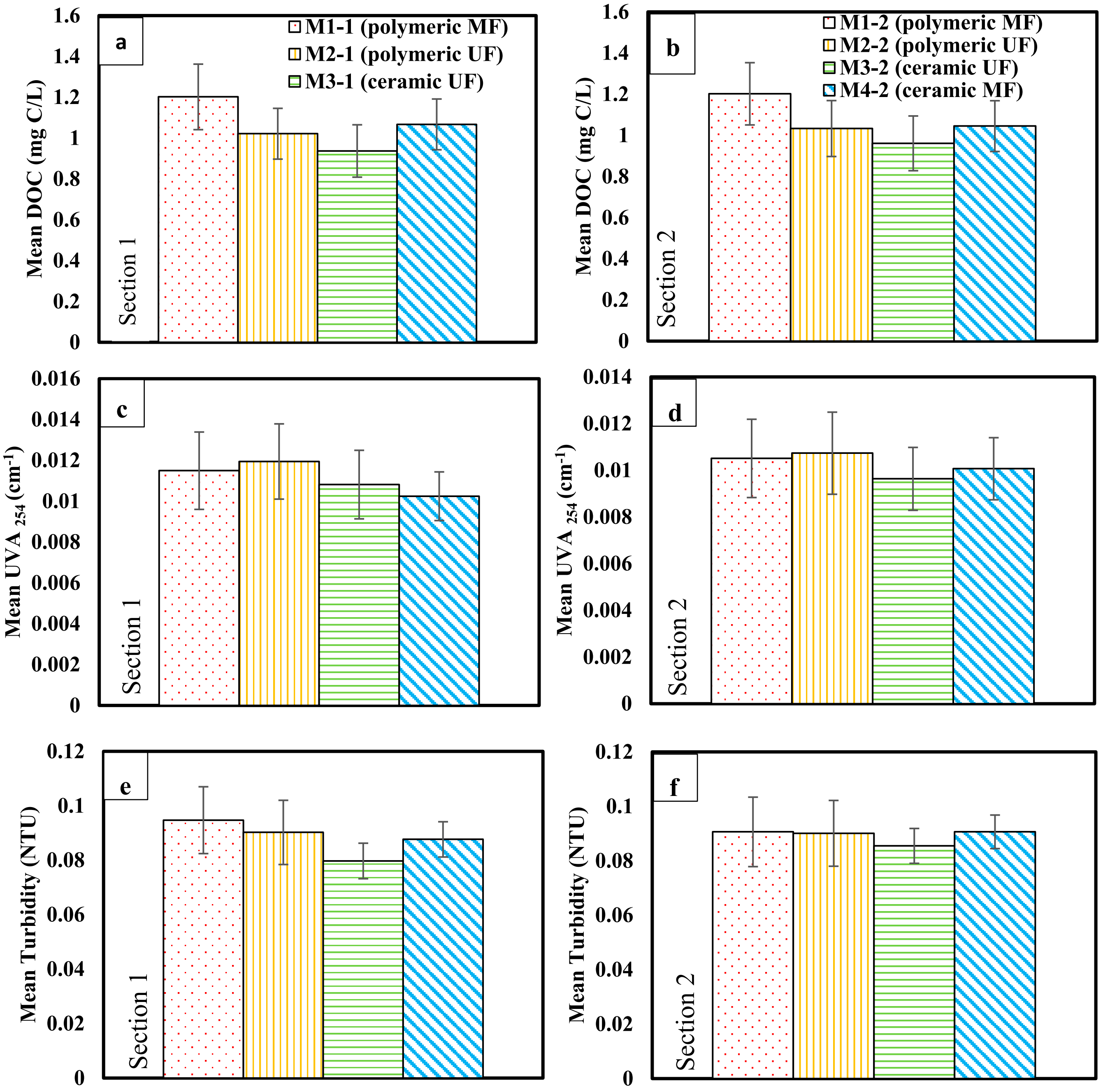

Figure S9. a, b) Mean DOC in section 1and section 2 days 1 – 68 c, d) Mean UVA_{254} in section 1 and section 2 days 1 – 68, e. f) Mean turbidity in section 1 and section 2 during the whole operation period. M1 (polymeric 0.1 µm MF), M2 (polymeric 0.03 µm UF), M3 (ceramic 300 kDa UF), and M4 (Lab-made ceramic MF). The error bars show 95% confidence intervals.
